# Lipidomic analysis of *Porphyromonas gingivalis* reveals novel glycerol bisphosphoceramide, phosphatidyl-, and phosphoglycerol dipeptide lipid families

**DOI:** 10.1016/j.jlr.2023.100470

**Published:** 2023-11-02

**Authors:** Brian A. Kleiboeker, Cheryl Frankfater, Mary E. Davey, Fong-Fu Hsu

**Affiliations:** 1 Mass Spectrometry Resource, Division of Endocrinology, Metabolism, and Lipid Research, Department of Medicine, Washington University School of Medicine, St. Louis, MO, USA; 2 Department of Microbiology, The Forsyth Institute, Cambridge, MA, USA

**Keywords:** bacterial lipids, bisphosphorylceramide glycerol, lipidomics, sphingolipids, peptidolipids, tandem mass spectrometry

## Abstract

*Porphyromonas gingivalis*, like other members of the phylum Bacteroidetes (synonym Bacteroidota), synthesizes several classes of dihydroceramides and peptidolipids. Using a similar strategy as that recently used to delimit the lipidome of its close relative *Bacteroides fragilis*, we applied linear ion trap multiple-stage mass spectrometry (linear ion trap MS^n^) with high-resolution mass spectrometry, to structurally characterize the complete lipidome of *P. gingivalis* and compare it to *B. fragilis*. This analysis discovered that the *P. gingivalis* lipidome consists of several previously unidentified lipid families, including dihydroceramide-1-phosphophate, acylated dihydroceramide-1-phosphophate, phosphoglycerol glycylserine lipid, and bis(phosphodihydroceramide) glycerol. Interestingly, we also found a novel sphingolipid family containing a polyunsaturated long–chain base, and a new lipoglycylserine phosphatic acid containing unsaturated acyl chains not reported for the lipid family. The comprehensive coverage of the lipidome of *P. gingivalis* conducted in this study has revealed more than 140 lipid species including several novel lipids in over 20 lipid families/subfamilies.

*Porphyromonas gingivalis* is a Gram-negative, oral anaerobe belonging to the phylum Bacteroidetes (1). This bacterium is strongly associated with development of destructive periodontal disease in adults (2, 3, 4), and infections with this bacterium are also correlated with a variety of systemic diseases, including atherosclerosis-associated cardiovascular diseases and Alzheimer’s disease (AD) (5, 6, 7, 8, 9, 10, 11). *P. gingivalis* synthesizes several classes of dihydroceramides (DHCs), including phosphoglycerol DHC (PG DHC), phosphoethanolamine DHC (PE DHC), as well as lipopeptides, including glycine lipid (G-lipid), lipoglycylserine (GS-lipid), and lipoglycylserine phosphatidic acid (GS-PA). At least one of these lipid classes have been shown to promote proinflammatory secretory reactions in gingival fibroblasts as well as alter fibroblast morphology in culture (12, 13, 14). Purified G- and GS-lipid were shown to promote toll-like receptor 2-dependent TNF-α release from bone marrow macrophages, and activate human embryonic kidney cells through toll-like receptor 2 and TLR6 but not TLR1 (15), and purified PG DHC has been shown to induce apoptosis, hence DHCs are thought to be important virulence determinants of *P. gingivalis* (16, 17, 18). In addition, recent studies have shown that synthesis of sphingolipids (SLs) by *P. gingivalis* is central to its ability to evade the host inflammatory response via the production of SL-containing outer membrane vesicles (19, 20, 21), indicating that the ratio of membrane lipids is important for homeostasis. Lastly, cell surface virulence determinants from *P. gingivalis* and *Bacteroides fragilis* have been detected in human AD brains (22, 23), and it has been hypothesized that infections with *P. gingivalis* or *B. fragilis* play a role in AD pathogenesis (23, 24).

DHC dihydrosphingosine (contains a unique methyl side chain (iso and anteiso) 17-, 18-, or 19-carbon sphinganine) base, to which a major iso-17:0 (3-OH) FA is linked to the 2-amino group. In PG DHC and PE DHC, an additional iso-15:0 FA can also be linked by an ester bond (“piggy back”) to the hydroxyl group of the 3-hydroxy 17:0-FA to form a 3-O-acyl- PG DHC (acylated-PG DHC) (25) and 3-O-acyl PE DHC (acylated-PE DHC), respectively (see Supporting material for identification). Structural characterization of lipid classes including PG DHC, PE DHC, serine dipeptide, and diacylated phosphoserine-glycine lipodipeptide specific to *P*. *gingivalis* was previously described by Nichols *et al.* (13, 14, 26). They applied chromatographic separation to isolate the lipid families, combined with chemical reactions, GC/MS, LC/MS, and NMR spectroscopic analyses to identify the structures. However, a complete lipid profile of *P. gingivalis* has not been previously reported. Recently, we reported the lipidomic analysis of *B. fragilis*, *Bacteroides vulgatus*, *Bacteroides thetaiotaomicron*, and *Bacteroides ovatus* in *Bacteroides* genus, which belong to the Bacteroidetes phylum same as *P. gingivalis*, and found several novel SL subfamilies (27). In light of the fact that these bacteria are highly related, we undertook a similar lipidomic analysis of *P. gingivalis* applying linear ion trap (LIT) multiple-stage MS (MS^n^) with high-resolution MS (HRMS) to profile the entire lipidome, revealing a lipidome including several lipid families that have not been previously reported.

## Materials and Methods

### Chemicals

All solvents in HPLC grade and other chemicals were purchased from Thermo Fisher Scientific (Waltham, MA USA). AMP+ MS Kit was purchased from Cayman Chemical Co (Ann Arbor, MI USA).

### Bacteria strains, cell growth, and lipid extraction

*P. gingivalis* strain W83 was grown on agar plates containing Trypticase Soy Broth (Becton, Dickinson and Company, Franklin Lakes, NJ, USA) supplemented with 5 μg ml^−1^ hemin, 1 μg ml^−1^ menadione, and 5% defibrinated sheep blood (Northeast Laboratory Services, Winslow, ME, USA) and incubated at 37°C in an anaerobic chamber (Coy Lab Products, Grass Lake, MI, USA) with an atmosphere containing 5% hydrogen, 10% carbon dioxide, and 85% nitrogen. Planktonic cultures of *P. gingivalis* were grown in Tryptic Soy Broth (TSB) medium (without dextrose) (Becton, Dickinson and Company, Franklin Lakes, NJ, USA) supplemented with 5 μg ml^−1^ hemin and 1 μg ml^−1^ menadione (TSBHK).

Planktonic cultures were grown, normalized, and extracted as described previously (28). In brief, *P. gingivalis* was inoculated into TSBHK, grown for 24 h, and then diluted into fresh TSBHK. Once the cultures reached exponential phase, they were normalized to an *A*_600_ of 1.0 and 1 ml of each culture was removed from the anaerobic chamber and centrifuged. The pellets were dissolved in chloroform/methanol/water (1.33:2.67:1, vol/vol/vol, 4 ml) as described previously (26). The mixture was vortexed at 15-min intervals for 2 h and then supplemented with 0.75 ml of chloroform and 0.75 ml of a buffer comprised of 2 N KCl and 0.5 N K_2_HPO_4_. The mixture was briefly vortexed, and centrifuged (2,000 *g*) at 20°C for 4 h. The lower organic phase was removed and dried under nitrogen. Lipid samples were dissolved in neutral HPLC solvent (hexane/isopropanol/water, 6:8:0.75, vol/vol/vol), the samples were centrifuged at 2,500  *g* for 10 min, and the supernatants were removed for analysis.

### Mass spectrometry

Both high-resolution (R = 100,000 at *m/z* 400) and low-energy collision-induced dissociation (CID) LIT MS^n^ analyses were conducted on a Thermo Fisher Scientific (San Jose, CA) LTQ Orbitrap Velos MS with Xcalibur operating system. Lipid extracts were dissolved in 1% NH_4_OH in methanol and infused or injected (via a loop) onto the ESI source and analyzed in the negative-ion mode. The skimmer of the source was set at ground potential, the electrospray needle was set at 4 kV, and temperature of the heated capillary was 300°C. The automatic gain control of the ion trap was set to 5 × 10^4^, with a maximum injection time of 50 ms. Helium was used as the buffer and collision gas at a pressure of 1 × 10^−3^ mbar (0.75 mTorr). The MS^n^ experiments were carried out with an optimized relative collision energy ranging from 25% to 45%, an activation q value of 0.25, and an activation time of 10 ms that leave minimal residual precursor ions with abundance around 20%. The mass selection window for the precursor ions was set at 1 Da wide to admit the monoisotopic ion to the ion trap (for CID) for unit resolution detection in the ion trap or HR accurate mass detection in the Orbitrap mass analyzer. Mass spectra were accumulated in the profile mode, typically for 2–10 min for MS^n^ spectra (n = 2, 3, 4).

### PtO_2_/H_2_ hydrogenation

For further insight into the structure of the polyunsaturated LCB substituent in glycerol phosphoryl ceramide (GPC) lipids, GPC fraction (c.a. 10 ug in 500 uL methanol) isolated by aminopropyl Sepak column as described previously (27) was placed in a tube, and 15 mg PtO_2_ was added, vortexed, and a stream of H_2_ was bubbled into the slurry at room temperature for 30 min. After reaction, the tube was centrifuged, and the methanol layer was transferred to another vial, and injected into mass spectrometer for HR ESI/MS analysis.

### Acid hydrolysis, free (FA) extraction, preparation of FA-N-(4-aminomethylphenyl)pyridinium derivatives, and tandem mass spectrometric analysis of FA-AMPP derivative for characterization of the FA substituents of the molecules

See Supplementary Material 2.

### Characterization of acylated PE-DHC by LIT MS^n^

See Supplementary Material 2.

### Nomenclature

The designations and abbreviations previously used for ceramides were adapted. Ceramides are abbreviated in the form of dLCB/FA, with d denoting a dihydroxy long–chain base (LCB), and FA referring to a fatty acid. The C_20_-chain length LCB with four unsaturated bonds is designated as d20:4-LCB, and the saturated C_20_-chain LCB is designated as d20:0-LCB. Fatty acyl moieties with or without hydroxyl substituent were denoted as hFA or nFA, respectively. Therefore, ceramides with β-hydroxyl fatty acyl substituent and C_20_-chain length LCB without and with four unsaturated bonds are designated as d20:0/βhFA-Cer and d20:4/βhFA-Cer, respectively, where the former is also named as DHC. For PG DHC (also named "DHC PG", or glycerol phosphoryl ceramide "GPC"), for example, with N-iso-17:0(3-OH) FA and 19:0-LCB is designated as d19:0/βh17:0-PG Cer. If, for example, an additional iso-15:0 FA is ester linkage (“piggy back”) to the hydroxyl group of the 3-hydroxy 17:0-FA, the acylated PG DHC is designated as d19:0/15:0-βh17:0-PG Cer (a substituted PG DHC in the literature (26)). Similar abbreviations are applied to phosphoryl-1-DHC (DHC-1-P), (PE DHC or ethanolamine phosphoryl ceramide (EPC)), and phosphorylserine DHC (PS DHC or SPC). The designation of the fragment ions is according to the previously published literature (29, 30).

## Results

We profiled the lipids extracted from *P. gingivalis* cells by high resolution (R = 100,000 at *m/z* 400) ESI MS scan in the negative-ion mode via loop injection, similar to the methodology previously used for *B. fragilis* (27). Accurate mass measurements permit extraction of elemental composition of the molecules, and when combined with MS^n^ that allows further insight into the fragmentation processes readily afford accurate assignments of the lipid structure and the entire lipid repertoire can be depicted (Table 1) (See supplemental Fig. S1A for HR full-scan ESI-MS).

**Table 1:** The lipid repertoire of *Porphyromona gingivalis* obtained by LIT MSn with high-resolution MS

m/z	Intensity	Relative	Theo. Mass	Deviation	RDB Equiv.	Composition	Lipid family/Subfamily	Assigned structures^a^				
[M – H]^-^		%	Da	mDa				Major	Minor isomers			
540.5000	3314.3	0.032	540.4997	0.30	1.5	C33 H66 O4 N	Cer(h33:0)	d17:0/βh16:0-Cer				
554.5154	17914.6	0.170	554.5154	0.00	1.5	C34 H68 O4 N	Cer(h34:0)	d17:0/βh17:0; d18:0/βh16:0 Cer				
568.5312	38476.7	0.366	568.5311	0.10	1.5	C35 H70 O4 N	Cer(h35:0)	d18:0/βh17:0-Cer				
582.5468	31477.1	0.299	582.5466	0.20	1.5	C36 H72 O4 N	Cer(h36:0)	d19:0/βh17:0-Cer				
592.3986	34913.6	0.330	592.3984	0.18	2.5	C30 H59 O8 N P	PE(25:0)	nc				
606.4142	259844.8	2.470	606.4140	0.21	2.5	C31 H61 O8 N P	PE(26:0)	11:0/15:0				
620.4300	1263816.4	12.040	620.4297	0.29	2.5	C32 H63 O8 N P	PE(27:0)	12:0/15:0				
634.4456	5052524	48.120	634.4453	0.27	2.5	C33 H65 O8 N P	PE(28:0)	15:0/13:0	14:0/14:0			
648.4612	2843879.8	27.080	648.4610	0.25	2.5	C34 H67 O8 N P	PE(29:0)	14:0/15:0-PE	16:0/13:0			
662.4768	6473839	61.660	662.4766	0.17	2.5	C35 H69 O8 N P	PE(30:0)	15:0/15:0-PE				
676.4924	943019.6	8.980	676.4923	0.16	2.5	C36 H71 O8 N P	PE(31:0)	16:0/15:0-PE				
690.5081	129588.6	1.230	690.5079	0.13	2.5	C37 H73 O8 N P	PE(32:0)	17:0/15:0-PE	16:0/16:0			
704.5235	180117.8	1.720	704.5236	−0.05	2.5	C38 H75 O8 N P	PE(33:0)	18:0/15:0-PE	17:0/16:0-PE			
664.4195	86295.1	0.820	664.4195	0.04	3.5	C33 H63 O10 N P	PS(27:0)	14:0/13:0-PS				
678.4353	298026.4	2.840	678.4352	0.14	3.5	C34 H65 O10 N P	PS(28:0)	15:0/13:0-PS				
692.4510	205558.8	1.960	692.4508	0.12	3.5	C35 H67 O10 N P	PS(29:0)	14:0/15:0-PS	16:0/13:0			
706.4666	243945.7	2.320	706.4665	0.15	3.5	C36 H69 O10 N P	PS(30:0)	15:0/15:0-PS				
720.4823	101222.9	0.960	720.4821	0.18	3.5	C37 H71 O10 N P	PS(31:0)	16:0/15:0-PS				
342.2651	3101.9	0.140	342.2650	0.12	2.5	C19 H36 O4 N	G(17:0)	βh17:0-G				
538.4483	3563.1	0.150	538.4477	0.60	3.5	C32 H60 O5 N	acyl-G(30:0)	15:0-βh15:0-G				
552.4635	33,391.1	1.430	552.4633	0.16	3.5	C33 H62 O5 N	acyl-G(31:0)	15:0-βh16:0-G				
566.4793	22014.7	0.940	566.4790	0.29	3.5	C34 H64 O5 N	acyl-G(32:0)	15:0-βh17:0-G				
580.4948	6839.3	0.13	580.4946	0.16	3.5	C35 H66 O5 N	acyl-G(h33:0)	nc				
594.5105	1297	0.03	594.5103	0.16	3.5	C36 H68 O5 N	acyl-G(h34:0)	nc				
568.4584	5162.5	0.2	568.4583	0.15	3.5	C33 H62 O6 N	acyl-S(30:0)	13:0/Bh17:0-S	12:0/Bh18:0-S	11:0/Bh19:0-S		
582.4740	9432.3	0.400	582.4739	0.12	3.5	C34 H64 O6 N	acyl-S(31:0)	15:0-βh16:0-S				
596.4898	110198.7	4.710	596.4896	0.19	3.5	C35 H66 O6 N	acyl-S(32:0)	15:0-βh17:0-S				
610.5055	17382.6	0.3	610.5052	0.28	3.5	C36 H68 O6 N	acyl-S(33:0)	nc				
415.2814	44439.9	0.420	415.2814	0.04	3.5	C21 H39 O6 N2	GS(h16:0)	nc				
429.2972	600968.1	5.720	429.2970	0.16	3.5	C22 H41 O6 N2	GS(h17:0)	βh17:0-GS				
611.4643	70389.5	0.670	611.4641	0.18	4.5	C34 H63 O7 N2	acyl-GS(h29:0)	nc				
625.4800	307224.6	2.930	625.4797	0.27	4.5	C35 H65 O7 N2	acyl-GS(h30:0)	13:0-βh17:0-GS	14:0/βh16:0-GS	15:0/βh15:0-GS		
639.4956	1622630.4	15.450	639.4954	0.24	4.5	C36 H67 O7 N2	acyl-GS(h31:0)	15:0-βh16:0-GS	14:0/βh17:0-GS			
653.5112	7074778	67.380	653.5110	0.18	4.5	C37 H69 O7 N2	acyl-GS(h32:0)	15:0-βh17:0-GS				
667.5268	91666.3	0.870	667.5267	0.10	4.5	C38 H71 O7 N2	acyl-GS(h33:0)	16:0/βh17:0-GS	15:0/βh18:0-GS			
681.5424	199391	0.18	681.5423	0.08	4.5	C39 H73 O7 N2	acyl-GS(34:0)	nc				
695.5579	11165.7	0.01	695.5580	0.08	4.5	C40 H75 O7 N2	acyl-GS(35:0)	nc				
793.4981	14724.3	0.140	793.4985	−0.42	4.5	C39 H74 O12 N2 P	acyl-GS-PG(h31:0)	nc				
807.5141	135032.7	1.282	807.5141	−0.03	4.5	C40 H76 O12 N2 P	acyl-GS-PG(h32:0)	15:0-βh17:0-GS-PG				
821.5306	1354.8	0.013	821.5298	0.85	4.5	C41 H78 O12 N2 P	acyl-GS-PG(h33:0)	nc				
634.4816	121094	1.150	634.4817	−0.13	1.5	C34 H69 O7 N P	Cer-1-P(h34:0)	d17:0/βh17:0-Cer-1-P	d18:0/βh16:0-Cer-1-P			
648.4975	202992.8	1.930	648.4974	0.10	1.5	C35 H71 O7 N P	Cer-1-P(h35:0)	d18:0/βh17:0-Cer-1-P;	d19:0/βh16:0-Cer-1-P			
662.5132	194152.6	1.850	662.5130	0.20	1.5	C36 H73 O7 N P	Cer-1-P(h36:0)	d19:0/βh17:0-Cer-1-P				
676.5288	115399	1.100	676.5287	0.09	1.5	C37 H75 O7 N P	Cer-1-P(h37:0)	d20:0/βh17:0-Cer-1-P				
844.6801	46503	0.440	844.6801	0.04	2.5	C48 H95 O8 N P	acyl-Cer-1-P(h48:0)	nc				
858.6957	130271.1	1.240	858.6957	−0.01	2.5	C49 H97 O8 N P	acyl-Cer-1-P(h49:0)	d17:0/15:0-βh17:0-Cer-1-P	d18:0/15:0-βh16:0-Cer-1-P			
872.7114	118626.5	1.130	872.7114	0.00	2.5	C50 H99 O8 N P	acyl-Cer-1-P(h50:0)	d18:0/15:0-βh17:0-Cer-1-P				
886.7272	43469	0.410	886.7270	0.15	2.5	C51 H101 O8 N P	acyl-Cer-1-P(h51:0)	nc				
721.5140	83009.6	0.790	721.5137	0.31	2.5	C37 H74 O9 N2 P	SPC(h34:0)	nc				
735.5296	185567.3	1.770	735.5294	0.24	2.5	C38 H76 O9 N2 P	SPC(h35:0)	d18:0/βh17:0-SPC				
749.5454	199096.3	1.900	749.5450	0.33	2.5	C39 H78 O9 N2 P	SPC(h36:0)	d19:0/βh17:0-SPC				
763.5608	110346.2	1.050	763.5607	0.15	2.5	C40 H80 O9 N2 P	SPC(h37:0)	nc				
621.4617	89581.5	0.850	621.4613	0.41	1.5	C32 H66 O7 N2 P	EPC(h30:0)	d13:0/βh17:0-EPC				
635.4771	303383.1	2.890	635.4770	0.18	1.5	C33 H68 O7 N2 P	EPC(h31:0)	d14:0/βh17:0-EPC				
649.4928	881656.7	8.400	649.4926	0.17	1.5	C34 H70 O7 N2 P	EPC(h32:0)	d15:0/βh17:0-EPC				
663.5084	1708532.9	16.270	663.5083	0.16	1.5	C35 H72 O7 N2 P	EPC(h33:0)	d17:0/βh16:0-EPC	d18:0/βh15:0-EPC	d16:0/βh17:0-EPC		
677.5241	4425368	42.150	677.5239	0.15	1.5	C36 H74 O7 N2 P	EPC(h34:0)	d18:0/βh16:0-EPC	d17:0/βh17:0-EPC			
691.5397	6604419	62.900	691.5396	0.10	1.5	C37 H76 O7 N2 P	EPC(h35:0)	d18:0/βh17:0-EPC	d19:0/βh16:0-EPC			
705.5553	3074976.8	29.290	705.5552	0.11	1.5	C38 H78 O7 N2 P	EPC(h36:0)	d19:0/βh17:0-EPC				
719.5710	837055.9	7.970	719.5709	0.12	1.5	C39 H80 O7 N2 P	EPC(h37:0)	d21:0/βh16:0-EPC				
683.4773	299954.3	2.860	683.4770	0.30	5.5	C37 H68 O7 N2 P	EPC(h35:4)	nc				
697.4928	1239502.3	11.800	697.4926	0.18	5.5	C38 H70 O7 N2 P	EPC(h36:4)	d20:4/βh16:0-EPC				
711.5084	1699240.5	16.180	711.5083	0.12	5.5	C39 H72 O7 N2 P	EPC(h37:4)	d20:4/βh17:0-EPC				
887.7230	47138.7	0.450	887.7223	0.72	2.5	C50 H100 O8 N2 P	acyl-EPC(49:0)	nc				
901.7379	109177.9	1.040	901.7379	−0.01	2.5	C51 H102 O8 N2 P	acyl-EPC(50:0)	d17:0/15:0-βh17:0-EPC	d18:0/15:0-βh16:0-EPC	d19:0/15:0-βh15:0-EPC		
915.7537	132681.6	1.260	915.7536	0.11	2.5	C52 H104 O8 N2 P	acyl-EPC(51:0)	d18:0/15:0-βh17:0-EPC				
929.7693	64680.2	0.620	929.7692	0.10	2.5	C53 H106 O8 N2 P	acyl-EPC(52:0)	d19:0/15:0-βh17:0-EPC				
666.4716	79139	0.750	666.4715	0.10	1.5	C34 H69 O9 N P	GPC(h31:0)	nc				
680.4873	307289.6	2.930	680.4872	0.07	1.5	C35 H71 O9 N P	GPC(h32:0)	nc				
694.5029	591308.5	5.630	694.5028	0.07	1.5	C36 H73 O9 N P	GPC(h33:0)	d16:0/βh17:0-GPC	d17:0/βh16:0-GPC	d18:0/βh15:0-GPC		
708.5186	1927814	18.360	708.5185	0.15	1.5	C37 H75 O9 N P	GPC(h34:0)	d17:0/βh17:0-GPC	d18:0/βh16:0-GPC			
722.5343	3559912.3	33.900	722.5341	0.11	1.5	C38 H77 O9 N P	GPC(h35:0)	d18:0/βh17:0-GPC	d19:0/βh16:0-GPC			
736.5499	2399642.5	22.850	736.5498	0.12	1.5	C39 H79 O9 N P	GPC(h36:0)	d19:0/βh17:0-GPC				
750.5655	443368.5	4.220	750.5654	0.09	1.5	C40 H81 O9 N P	GPC(h37:0)	d20:0/βh17:0-GPC				
714.4717	119091.6	1.130	714.4715	0.15	5.5	C38 H69 O9 N P	GPC(h35:4)	d20:4/βh15:0-GPC				
728.4873	573457.2	5.460	728.4872	0.13	5.5	C39 H71 O9 N P	GPC(h36:4)	d20:4/βh16:0-GPC				
742.5030	1492663.4	14.220	742.5028	0.11	5.5	C40 H73 O9 N P	GPC(h37:4)	d20:4/βh17:0-GPC				
756.5187	42787.8	0.410	756.5185	−0.12	5.5	C41 H75 O9 N P	GPC(h38:4)	nc				
862.6543	94767.9	0.900	862.6543	−0.01	2.5	C47 H93 O10 N P	acyl-GPC(h44:0)	nc				
876.6700	289121.4	2.750	876.6699	0.04	2.5	C48 H95 O10 N P	acyl-GPC(h45:0)	15:0-d15:0/βh15:0-GPC	15:0-d17:0/βh13:0-GPC			
890.6856	672718.3	6.410	890.6856	0.00	2.5	C49 H97 O10 N P	acyl-GPC(h46:0)	15:0-d15:0/βh16:0-GPC	15:0-d17:0/βh14:0-GPC	15:0-d16:0/βh15:0-GPC	14:0-d15:0/βh17:0-GPC	14:0-d16:0/βh17:0-GPC
904.7013	1802231.3	17.160	904.7012	0.09	2.5	C50 H99 O10 N P	acyl-GPC(h47:0)	15:0-d15:0/βh17:0-GPC	15:0-d17:0/βh15:0-GPC	15:0-d18:0/βh14:0-GPC	15:0-d16:0/βh16:0-GPC	d19:0/14:0-βh16:0-GPC
918.7169	2740170.3	26.100	918.7169	0.09	2.5	C51 H101 O10 N P	acyl-GPC(h48:0)	d18:0/15:0-βh15:0-GPC	d17:0/15:0-βh16:0-GPC	d16:0/15:0-βh17:0-GPC	d17:0/14:0-βh17:0-GPC	
932.7325	6384290	60.800	932.7325	0.03	2.5	C52 H103 O10 N P	acyl-GPC(h49:0)	d17:0/15:0-βh17:0-GPC	d18:0/15:0-βh16:0-GPC	d19:0/15:0-βh15:0-GPC	d18:0/14:0-βh17:0-GPC	
946.7481	10137694	96.550	946.7482	−0.02	2.5	C53 H105 O10 N P	acyl-GPC(h50:0)	d18:0/15:0-βh17:0-GPC	d19:0/15:0-βh16:0-GPC	d17:0/15:0-βh18:0-GPC	d20:0/15:0-βh15:0-GPC	
960.7637	6035595.5	57.480	960.7638	−0.07	2.5	C54 H107 O10 N P	acyl-GPC(h51:0)	d19:0/15:0-βh17:0-GPC				
974.7794	1088848.3	10.370	974.7795	−0.08	2.5	C55 H109 O10 N P	acyl-GPC(h52:0)	d20:0/15:0-βh17:0-GPC	d21:0/15:0-βh16:0-GPC			
910.6544	53489.1	0.510	910.6543	0.15	6.5	C51 H93 O10 N P	acyl-GPC(h48:4)	nc				
924.6700	117443.8	1.120	924.6699	0.08	6.5	C52 H95 O10 N P	acyl-GPC(h49:4)	nc				
938.6856	486247.9	4.630	938.6856	0.09	6.5	C53 H97 O10 N P	acyl-GPC(h50:4)	d20:4/15:0-βh15:0-GPC				
952.7012	1309631.8	12.470	952.7012	0.03	6.5	C54 H99 O10 N P	acyl-GPC(h51:4)	d20:4/15:0-βh16:0-GPC	d20:4/14:0-βh17:0-GPC			
966.7167	2729351	25.990	966.7169	−0.15	6.5	C55 H101 O10 N P	acyl-GPC(h52:4)	d20:4/15:0-βh17:0-GPC				
980.7325	115134.3	1.100	980.7325	−0.05	6.5	C56 H103 O10 N P	acyl-GPC(h53:4)	nc				
994.7481	134879.7	1.280	994.7482	−0.10	6.5	C57 H105 O10 N P	acyl-GPC(h54:4)	d22:4/15:0-βh17:0-GPC	d19:0/18:4-βh17:0-GPC			
1031.7286	78989.4	0.750	1031.7282	0.45	5.5	C55 H104 O13 N2 P	GS-PA(h47:0)	βh17:0-GS-15:0/15:0-PA				
1071.7598	102209.2	0.970	1071.7595	0.35	6.5	C58 H108 O13 N2 P	GS-PA(h48:0)	βh17:0-GS-18:1/15:0-PA				
1185.8634	27354.9	0.106	1185.8639	−0.53	6.5	C65 H122 O14 N2 P	acyl-GS-PA(h57:0)	15:0/Bh16:0-GS-13:0/13:0-PA	15:0/Bh15:0-GS-14:0/13:0-PA			
1199.8796	163605.3	0.635	1199.8796	0.05	6.5	C66 H124 O14 N2 P	acyl-GS-PA(h58:0)	15:0/Bh16:0-GS-14:0/13:0-PA	14:0/Bh16:0-GS-15:0/13:0-PA	13:0/Bh16:0-GS-14:0/15:0-PA		
1213.8953	523358.5	2.032	1213.8952	0.05	6.5	C67 H126 O14 N2 P	acyl-GS-PA(h59:0)	15:0/Bh16:0-GS-15:0/13:0-PA	14:0/Bh16:0-GS-14:0/15:0-PA	15:0/Bh17:0-GS-14:0/13:0-PA		
1227.9109	1277241	4.960	1227.9109	0.04	6.5	C68 H128 O14 N2 P	acyl-GS-PA(h60:0)	15:0/Bh16:0-GS-14:0/15:0-PA	14:0/Bh16:0-GS-15:0/15:0-PA	15:0/Bh17:0-GS-15:0/13:0-PA		
1241.9264	1112966.4	4.322	1241.9265	−0.07	6.5	C69 H130 O14 N2 P	acyl-GS-PA(h61:0)	15:0/Bh16:0-GS-15:0/15:0-PA	16:0/Bh16:0-GS-14:0/15:0-PA			
1255.9420	1954418.9	7.590	1255.9422	−0.18	6.5	C70 H132 O14 N2 P	acyl-GS-PA(h62:0)	15:0/Bh17:0-GS-15:0/15:0-PA	15:0/Bh16:0-GS-16:0/15:0-PA			
1269.9587	136135.7	1.300	1269.9578	0.90	6.5	C71 H134 O14 N2 P	acyl-GS-PA(h63:0)	nc				
1239.9106	13785.6	0.053	1239.9109	−0.21	7.5	C69 H128 O14 N2 P	acyl-GS-PA(h61:1)	nc				
1253.9272	36948	0.143	1253.9265	0.68	7.5	C70 H130 O14 N2 P	acyl-GS-PA(h62:1)	nc				
1267.9426	77938.8	0.740	1267.9422	0.44	7.5	C71 H132 O14 N2 P	acyl-GS-PA(h63:1)	nc				
1281.9583	599401.6	2.328	1281.9578	0.46	7.5	C72 H134 O14 N2 P	acyl-GS-PA(h64:1)	15:0/βh16:0-GS-15:0/18:1-PA				
1295.9742	1298862.3	5.044	1295.9735	0.75	7.5	C73 H136 O14 N2 P	acyl-GS-PA(h65:1)	15:0/βh17:0-GS-15:0/18:1-PA				
1309.9899	297373.8	1.155	1309.9891	0.84	7.5	C74 H138 O14 N2 P	acyl-GS-PA(h66:1)	nc				
1324.0050	18460.3	0.072	1324.0048	0.22	7.5	C75 H140 O14 N2 P	acyl-GS-PA(h67:1)	nc				
1338.0198	24848.1	0.096	1338.0204	−0.59	7.5	C76 H142 O14 N2 P	acyl-GS-PA(h68:1)	nc				
1251.9109	8190.8	0.032	1251.9109	0.03	8.5	C70 H128 O14 N2 P	acyl-GS-PA(h62:2)	nc				
1279.9425	380659	1.478	1279.9422	0.31	8.5	C72 H132 O14 N2 P	acyl-GS-PA(h64:2)	15:0/βh16:0-GS-15:0/18:2-PA				
1293.9583	755818.2	2.935	1293.9578	0.5	8.5	C73 H134 O14 N2 P	acyl-GS-PA(h65:2)	15:0/βh17:0-GS-15:0/18:2-PA				
1307.9738	195323	0.759	1307.9735	0.29	8.5	C74 H136 O14 N2 P	acyl-GS-PA(h66:2)	nc				
1321.9873	3973.2	0.015	1321.9891	1.82	8.5	C75 H138 O14 N2 P	acyl-GS-PA(h67:2)	nc				
1336.0042	14006.7	0.054	1336.0048	−0.53	8.5	C76 H140 O14 N2 P	acyl-GS-PA(h68:2)	nc				
1434.1155	3030.2	0.012	1434.1143	1.20	8.5	C83 H154 O14 N2 P	acyl-GS-PA(h75:2)	nc				
1277.9269	125638	0.488	1277.9265	0.37	9.5	C72 H130 O14 N2 P	acyl-GS-PA(h64:3)	15:0/βh17:0-GS-14:0/18:3-PA	15:0/βh16:0-GS-15:0/18:3-PA			
1291.9427	285593.1	1.109	1291.9422	0.56	9.5	C73 H132 O14 N2 P	acyl-GS-PA(h65:3)	15:0/βh17:0-GS-15:0/18:3-PA				
1305.9594	75435.7	0.293	1305.9578	1.55	9.5	C74 H134 O14 N2 P	acyl-GS-PA(h66:3)	nc				
1319.9749	31321	0.122	1319.9735	1.44	9.5	C75 H136 O14 N2 P	acyl-GS-PA(h67:3)	nc				
1460.1299	2224.8	0.009	1460.1300	0.04	9.5	C85 H156 O14 N2 P	acyl-GS-PA(h77:3)	nc				
1233.8636	23218.6	0.090	1233.8639	−0.34	10.5	C69 H122 O14 N2 P	acyl-GS-PA(h61:4)	nc				
1247.8793	41190.2	0.160	1247.8796	−0.25	10.5	C70 H124 O14 N2 P	acyl-GS-PA(h62:4)	nc				
1275.9109	62861	0.244	1275.9109	0.03	10.5	C72 H128 O14 N2 P	acyl-GS-PA(h64:4)	nc				
1289.9266	150840.5	0.586	1289.9265	0.12	10.5	C73 H130 O14 N2 P	acyl-GS-PA(h65:4)	15:0/βh17:0-GS-18:4/15:0-PA				
1303.9425	118273.9	0.460	1303.9422	0.31	10.5	C74 H132 O14 N2 P	acyl-GS-PA(h66:4)	15:0/βh16:0-GS-15:0/20:4-PA				
1317.9588	210331.6	0.817	1317.9578	1.00	10.5	C75 H134 O14 N2 P	acyl-GS-PA(h67:4)	15:0/βh17:0-GS-15:0/20:4-PA				
1301.9273	102123.6	0.397	1301.9265	0.77	11.5	C74 H130 O14 N2 P	acyl-GS-PA(h66:5)	nc				
1315.9422	176,583.9	0.686	1315.9422	−0.05	11.5	C75 H132 O14 N2 P	acyl-GS-PA(h67:5)	15:0/βh17:0-GS-15:0/20:5-PA				
1329.9582	142,176.8	0.553	1329.9578	0.35	11.5	C76 H134 O14 N2 P	acyl-GS-PA(h68:5)	15:0/βh17:0-GS-18:4/18:1-PA	15:0/βh17:0-GS-16:0/20:5-PA	15:0/βh16:0-GS-15:0/22:5-PA		
1343.9744	58885.9	0.229	1343.9735	0.94	11.5	C77 H136 O14 N2 P	acyl-GS-PA(h69:5)	nc				
1357.9897	14314.2	0.056	1357.9891	0.63	11.5	C78 H138 O14 N2 P	acyl-GS-PA(h70:5)	nc				
1340.0129	107217	0.339	1340.0126	0.30	2.5	C72 H145 O15 N2 P2	Cer-PGP-Cer(h269:0)	d18:0/βh17:0-DHC-PGP-d17:0/βh17:0	d19:0/βh17:0-DHC-P-G-P-d18:0/βh15:0	d19:0/βh17:0-DHC-P-G-P-d17:0/βh16:0		
1354.0283	341764.3	1.082	1354.0282	0.10	2.5	C73 H147 O15 N2 P2	Cer-PGP-Cer(h270:0)	d18:0/βh17:0-DHC-PGP-d18:0/βh17:0	d19:0/βh17:0-DHC-P-G-P-d17:0/βh17:0	d19:0/βh17:0-DHC-P-G-P-d18:0/βh16:0		
1368.0439	631934.1	2.000	1368.0439	0.00	2.5	C74 H149 O15 N2 P2	Cer-PGP-Cer(h271:0)	d19:0/βh17:0-DHC-PGP-d18:0/βh17:0				
1382.0594	601635.3	1.904	1382.0595	−0.10	2.5	C75 H151 O15 N2 P2	Cer-PGP-Cer(h272:0)	d19:0/βh17:0-DHC-PGP-d19:0/βh17:0	d20:0/βh17:0-DHC-P-G-P-d18:0/βh17:0			
1396.0758	98397.9	0.311	1396.0752	0.60	2.5	C76 H153 O15 N2 P2	Cer-PGP-Cer(h273:0)	nc				
1359.9812	24001.7	0.076	1359.9813	−0.07	6.5	C74 H141 O15 N2 P2	Cer-PGP-Cer(h271:4)	nc				
1345.9657	4732.7	0.015	1345.9656	0.13	6.5	C73 H139 O15 N2 P2	Cer-PGP-Cer(h272:4)	nc				
1373.9968	81570.1	0.258	1373.9969	−0.07	6.5	C75 H143 O15 N2 P2	Cer-PGP-Cer(h273:4)	d18:0/βh17:0-DHC-PGP-d20:4/βh17:0				
1388.0126	107554.5	0.340	1388.0126	0.03	6.5	C76 H145 O15 N2 P2	Cer-PGP-Cer(h274:4)	d19:0/βh17:0-DHC-PGP-d20:4/βh17:0				
1402.0279	35742.2	0.113	1402.0282	−0.27	6.5	C77 H147 O15 N2 P2	Cer-PGP-Cer(h275:4)	nc				
1564.2273	3497.9	0.011	1564.2266	0.71	3.5	C87 H173 O16 N2 P2	acyl-Cer-pgp-Cer(h284:0)	nc				
1578.2422	15909.3	0.050	1578.2422	−0.03	3.5	C88 H175 O16 N2 P2	acyl-Cer-pgp-Cer(h285:0)	d18:0/βh17:0-DHC-PGP-d18:0/15:0-βh17:0				
1592.2580	23730.1	0.075	1592.2579	0.13	3.5	C89 H177 O16 N2 P2	acyl-Cer-pgp-Cer(h286:0)	d19:0/βh17:0-DHC-PGP-d18:0/15:0-βh17:0				
1606.2739	16655.1	0.053	1606.2735	0.39	3.5	C90 H179 O16 N2 P2	acyl-Cer-pgp-Cer(h287:0)	nc				
1584.1964	991.7	0.003	1584.1953	1.08	7.5	C89 H169 O16 N2 P2	acyl-Cer-pgp-Cer(h286:4)	d20:4/βh17:0-DHC-PGP-d18:0/15:0-βh17:0				
1598.2100	4356.9	0.014	1598.2109	−0.96	7.5	C90 H171 O16 N2 P2	acyl-Cer-pgp-Cer(h287:4)	nc				

Cer-1-P, ceramide-1-phosphate; EPC, ethanolamine phosphoryl ceramide; GPC, glycerol phosphoryl ceramide; GS, glycylserine; nc, not characterized; PA, phosphatic acid; PE, phosphoethanolamine; PG, phosphoglycerol; PS, phosphorylserine; SPC (or ∗PS DHC), serine phosphoryl ceramide.

a isomer abundance in the descending order.

### Characterization of GS-lipid, G-lipid, serine lipid, and a new GS-PG lipid family

**Fig. 1:**
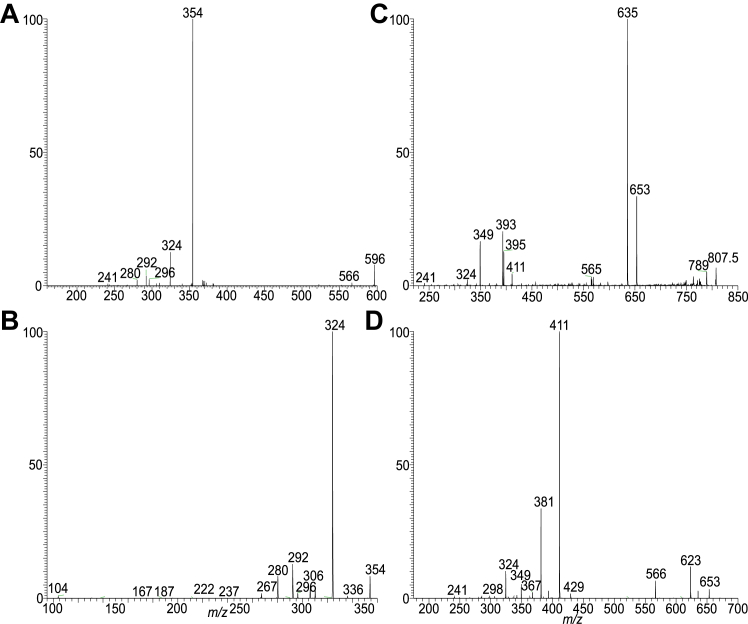
The LIT MS^2^ spectrum of the [M – H]^-^ ion of 15:0-βh17:0-Ser lipid at *m/z* 596 (A), its MS^3^ spectrum of *m/z* 354 (596 →354) (B); panel C is the MS^2^ spectrum of a new GS-PG lipid ion of *m/z* 807 (C) and its MS^3^ spectrum of the ion of *m/z* 653 (807 →653) (D) arising from 15:0-βh17:0-GS PG) in which a phosphoglycerol tail is likely attached to the -OH group of the serine residue. GS, glycylserine; PG, phosphoglycerol.

**Scheme 1:**
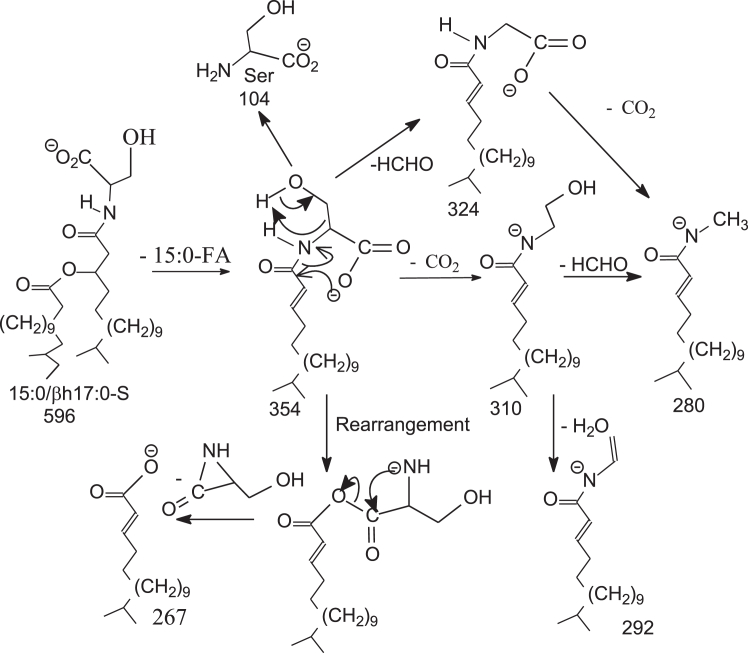
The fragmentation processes proposed for the [M – H]^-^ ion of 15:0/βh17:0-S at *m/z* 596.

The serine-glycine dipeptide lipids produced by *P. gingivalis* were previously defined as lipid 654 (31), which was detected at *m/z* 653 and a homologous ions at *m/z* 611, 625, and 677, as the [M – H]^-^ ions in the negative-ion mode. GS-lipids also contain ions at *m/z* 415 and 429, in which the acyl chain attached to the β-OH FA substituent is absent. G-lipids termed lipid 567 and lipid 342 consisted of similar FA substituents and have also been reported (15). In this study, serine lipids (S-lipids) were identified, which were seen at *m/z* 596.4898 (calculated C_35_H_66_O_6_N:596.4896) and *m/z* 582.4740 (calculated C_34_H_64_O_6_N: 582.4739) in the negative-ion mode, identical to those found in *B. fragilis* group (27). Structural characterization of these peptidolipids applying LIT MS^n^ is exemplified by HR MS^2^ on the S-lipid ion of *m/z* 596 (Fig. 1A), which gave rise to a major ion of *m/z* 354, arising from elimination of β-hydroxy C15-acyl chains as FA (15:0-FA) to form a N-C17:1-acyl-S. The MS^3^ spectrum of *m/z* 354 (596 →354; Fig. 1B) contained the ion at *m/z* 267 arising from loss of [Ser -H_2_O] (87 Da), consistent with the presence of *m/z* 104 representing a serine anion. The spectrum also contained a major ion at *m/z* 324 arising from loss of HCHO (Scheme 1), and an ion at *m/z* 280 from further loss of CO_2_. This fragmentation process was supported by the MS^4^ spectrum of *m/z* 324 (596 →354 →324; data not shown), which is dominated by ion of *m/z* 280. Loss of CO_2_ from *m/z* 354 also gave rise to *m/z* 310, which further dissociated to *m/z* 280 (310 – HCHO) and 292 (310 – H_2_O) by further losses of HCHO and H_2_O, respectively. The above results readily led to define a N-(3-pentadecanoyloxy) hepetadecanoyl serine (15:0-βh17:0-Ser) structure (Scheme 1).

**Scheme 2:**
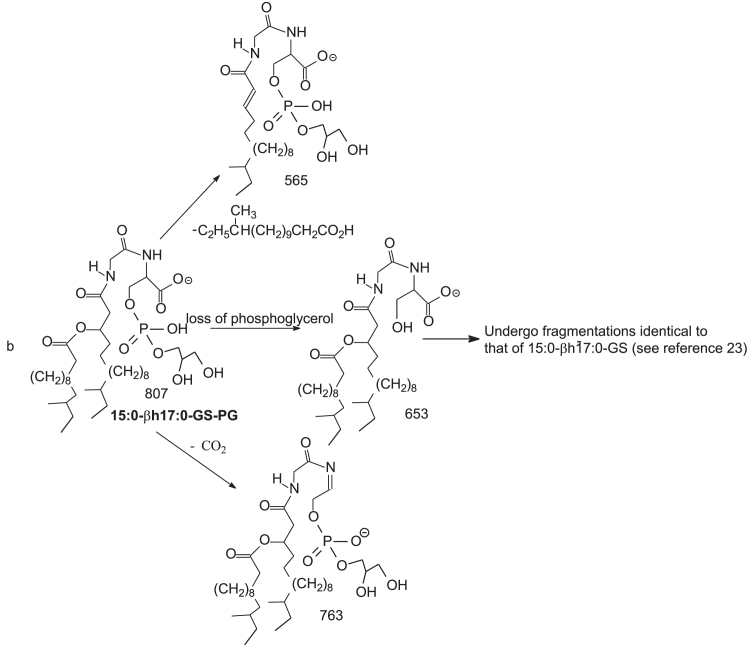
The fragmentation processes proposed for the [M – H]^-^ ion of 15:0-βh17:0-Cer-GS-PG at *m/z* 807 the 15L0- and 17:0-FA chains are in both iso/anteiso forms. GS, glycylserine; PG, phosphoglycerol.

Applying HRMS, we also found a minor new family seen at *m/z* 793.4981 (calculated C_39_H_74_O_12_N_2_P:793.4985), 807.5141 (calculated C_40_H_76_O_12_N_2_P: 807.5141), and 821.5294 (calculated C_41_H_78_O_12_N_2_P: 821.5298) (See supplemental Fig. S1B), which are C_3_H_7_O_5_P (154.0031 Da) heavier than the corresponding GS-lipid ions at 639.4954, 653.5110, and 667.5267. MS^2^ on the ion of *m/z* 807 (Fig. 1C) gave rise to ions at *m/z* 653 (loss of 154 Da) and 635 (653 – H_2_O) arising from loss of phosphoglycerol residue, indicating the attachment of a PG residue to the GS-lipid. The MS^3^ spectrum of the ion at *m/z* 653 (807 →653; Fig .1D) is identical to that observed for 15:0-βh17:0-GS (27), consistent with the notion that the molecule contains a GS-core structure. The results led us to define a 15:0-βh17:0-GS PG structure (GS-PG) in which the PG tail most likely attached to the -OH group of the serine residue (Scheme 2).

### Characterization of PE DHC (EPC), PG DHC (GPC), new PS DHC (SPC), and DHC-1-phosphate families

**Fig. 2:**
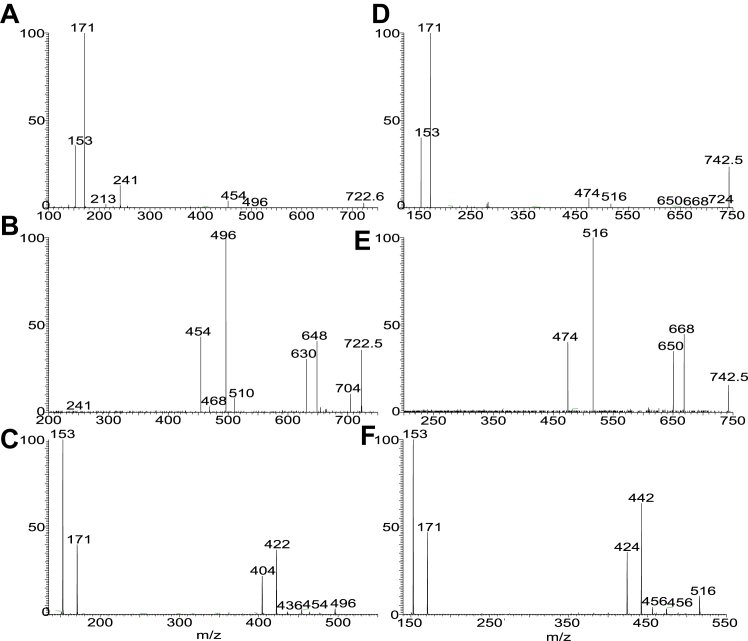
The MS^2^ spectrum of the [M – H]^-^ ion of *m/z* 722 obtained by higher collision CID (HCD; 70) (A), by collision-induced dissociation (CID; 35%) (B), and its MS^3^ spectrum at *m/z* 496 (722 → 496) (C), representing both a major d18:0/βh17:0-GPC and a d19:0/βh16:0-GPC minor isomer (from MS spectrum of *m/z* 510; not shown). The MS^2^ spectrum of the [M – H]^-^ ion at *m/z* 742 obtained by HCD (D), by LIT CID (E), and its MS^3^ spectrum of *m/z* 516 (742 → 516) (F) led to define a d20:4/βh17:0-GPC structure, a new GPC subfamily with a d20:4-LCB. GPC, glycerol phosphoryl ceramide; LCB, long-chain base; LIT, linear ion trap.

**Scheme 3:**
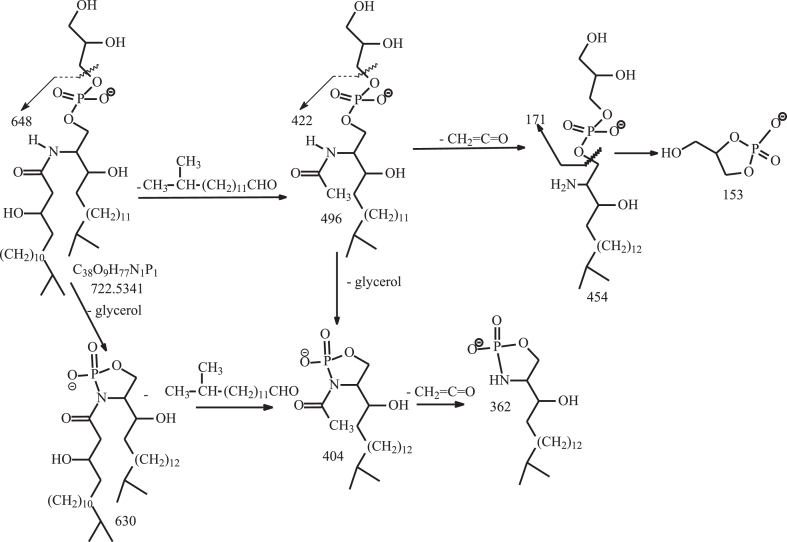
The fragmentation processes proposed for the [M – H ]^-^ ion of d18:0/βh17:0-Cer PG at *m/z* 722.5. PG, phosphoglycerol.

Nichols and colleagues identified and isolated the dominant members of DHC phospholipid family in *P. gingivalis*, including PG DHC, PE DHC, and 3-O-acylated PG DHC (substituted PG DHC) in which the LCB is fully saturated (i.e., sphinganine LCB) (26). However, PS DHC and DHC-1-phosphate lipid families that were detected in this study were not previously reported. We also found 3-O-acylated PE DHC and 3-O-acylated DHC-1-P lipid (Table 1). Interestingly, a novel PG ceramide and PE ceramide species with polyunsaturated LCB (d20:4-LCB) were also present (supplemental Fig. S1). The structures of these ceramide phospholipids (Table 1) were characterized by LIT MS^n^ approaches with high-resolution MS. For example, higher energy CID (HCD) on the [M – H]^-^ ion at *m/z* 722 (Fig. 2A) gave rise to major ions at 153 and 171, consistent with the notion that the molecule consists of PG head group (32). By contrast, the LIT MS^2^ spectrum of *m/z* 722 (Fig. 2B) contained ions at *m/z* 648 (loss of [glycerol – H_2_O]) and 630 (loss of glycerol), indicating the presence of glycerol head group (32), and the ions at *m/z* 496 arising from cleavage of the N-β-hydroxy-heptadecanoyl substituent as an aldehyde (loss of C_14_H_29_CHO; 226 Da) and at m/z 454 from further loss of an acetylene (CH_2_=CO) (Scheme 3). This fragmentation process is supported by the MS^3^ spectrum of the ion of *m/z* 496 (722 → 496; Fig. 2C), which contained ions of *m/z* 454, along with ions of *m/z* 422 (loss of [glycerol – H_2_O]; 74 Da) and 404 (loss of glycerol; 92 Da) and ions of *m/z* 171 and 153 that signify the presence of the PG head group (Scheme 3). The above structural information readily led to the assignment of a d18:0/βh17:0-GPC structure. The spectrum (Fig. 2B) also contained the ion of *m/z* 510, arising from the analogous loss of N-β-hydroxy-hexadecanoyl substituent as an aldehyde (loss of C_13_H_27_CHO; 212 Da), indicating the presence of a d19:0/βh16:0-GPC minor isomer. Similarly, the HCD MS^2^ spectrum of the [M – H]^-^ ion at *m/z* 742 (Fig. 2D), and CID MS^2^ spectrum of the ion at *m/z* 742 (Fig. 2E) contained ions of *m/z* 668 (742 – 74) and 650 (742 – 92) from loss of glycerol, and ions of *m/z* 171 and 153 (Fig. 2D) representing PG head group, together with *m/z* 516 (loss of C_14_H_29_CHO; 226 Da) indicating the presence of a βh17:0 substituent. The MS^3^ spectrum of *m/z* 516 (742 → 516; Fig. 2F) contained ions at *m/z* 442/424 (loss of glycerol), and ions at *m/z* 171 and 153; and the spectrum profile is similar to that of Fig. 2C. Taken together, the results indicated the presence of a d20:4/βh17:0-GPC, a new GPC subfamily with a d20:4-LCB.

**Fig. 3:**
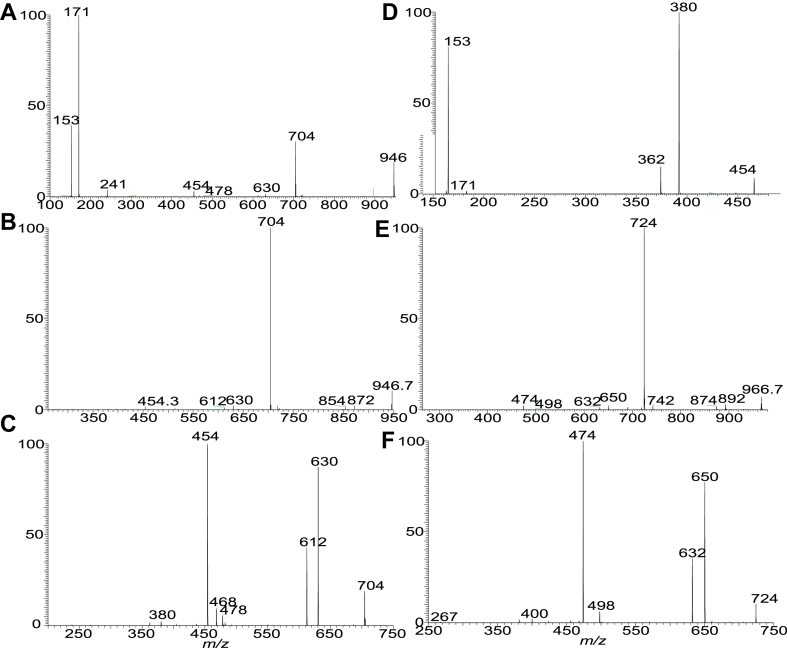
The MS^2^ spectrum of the [M – H]^-^ ion of d18:0/15:0-β17:0-GPC at *m/z* 946 obtained by HCD (A), by LIT CID (B) and its MS^3^ spectrum of *m/z* 704 (946 →704) (C), MS^4^ spectrum of *m/z* 454 (946 →704 →454) (D) from a acylated GPC lipid; The MS^2^ spectrum of *m/z* 966 obtained by LIT CID (E) and its MS^3^ spectrum of *m/z* 724 (panel F) led to define a d20:4/15:0-β17:0-GPC structure, an acylated GPC species with d20:4-LCB. CID, collision-induced dissociation; GPC, glycerol phosphoryl ceramide; HCD, higher energy CID; LIT, linear ion trap; LCB, long-chain base.

**Scheme 4:**
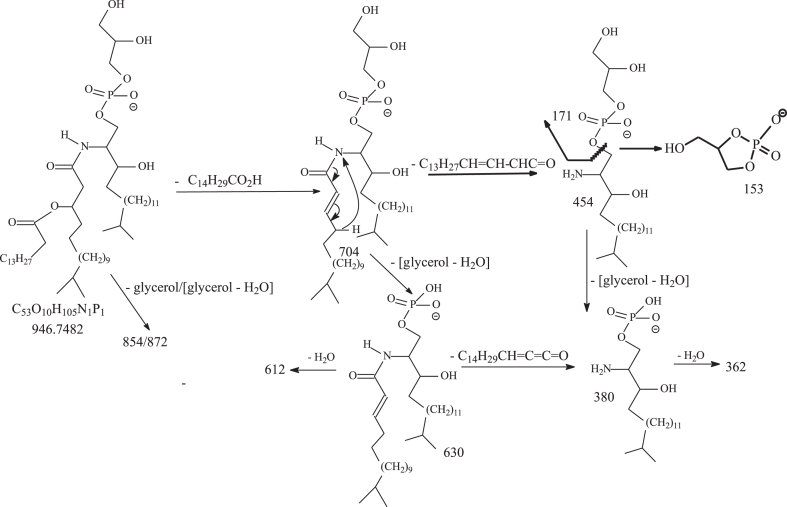
The fragmentation processes proposed for the [M – H]^-^ of d18:0/15:0-βh17:0-GPG at *m/z* 946.7.

ESI high-resolution mass measurement also showed the presence of the ion series of *m/z* 890.6856, …, 946.7481, 960.7637, and 974.7794 (supplemental Fig. S1D) (Table 1), which are 224 Da (C_13_H_27_CH=CO) heavier than the ceramide PG lipids seen at *m/z* 666.4716, 680.4873, .. , 736.5499, and 756.5187, indicating the presence of an acylated Cer-PG family in which a 15:0-fatty acyl group is ester bonded to the 3-hydroxy fatty acyl chain. High-resolution CID MS^2^ spectrum of *m/z* 946 (Fig. 3A) and HCD MS^2^ spectrum of *m/z* 946 (Fig. 3B) indicate that the major fragment ion at *m/z* 704 arose from elimination of the 15:0-fatty acyl substituent (loss of 15:0-FA) to form an d18:0/N-α,β-unsaturated 17:1-GPC (d18:0/17:1-GPC) (Scheme 4). The MS^3^ spectrum of the ion of *m/z* 704 (946 →704; Fig. 3C) is dominated by ions at *m/z* 630 (loss of [glycerol – H_2_O]) and 612 (loss of glycerol) arising from loss of the glycerol head group, consistent with the GPC structure. The spectrum also contained *m/z* 454 arising from further loss of the 17:1-fatty acyl ketene (loss of C_14_H_29_CH=C=C=O; 250 Da), along with ions at *m/z* 362/380 from further loss of the glycerol head (from *m/z* 454). This latter fragmentation process is supported by the MS^4^ spectrum of *m/z* 454 (946 →704 →454; Fig. 3D). The above results readily identify a d18:0/15:0-β17:0-GPC structure. A similar acyl-GPC subfamily consisting of the same fatty acyl substituent but with d20:4-LCB were also observed at m/z 952.7012, 966.7167, 980.7325, and 994.7481. For example, MS^2^ on the ion at m/z 966 (Fig. 3E) gave rise to a major ion at m/z 724 arising from loss of 15:0-FA. The MS^3^ spectrum of *m/z* 724 (966 →724; Fig. 3F) contained the major ion at *m/z* 474 arising from similar loss of the 17:1-fatty acyl ketene, indicating that the molecule contained the same N-15:0-β17:0-fatty acyl chain attached to the d20:4-LCB. The results are in accord with the earlier notion of the presence of the GPC with polyunsaturated LCB moieties.

To provide further insight into the unsaturation status of the LCB of the molecules (e.g., d20:4-LCB), we applied HRMS analysis on the reaction product of the above lipid families after hydrogenation with PtO_2_/H_2_ at room temperature. The high-resolution ESI mass spectrum showed that the ions at *m/z* 742 and 966, the speculated d20:4/βh17:0-GPC and d20:4/15:0-βh17:0-GPC, respectively, vanished; while new ions appeared at *m/z* 748 and 750 that are 6 and 8 hydrogens heavier (supported by high-resolution mass measurements), than *m/z* 742; and ions at *m/z* 972 and 974 that are also 6 and 8 hydrogens heavier than *m/z* 966, respectively, were observed. The hydrogenation of 3 and 4 alkene bonds is consistent with the earlier notion of the presence of GPC lipids with d20:4-LCB, a polyunsaturated alkenyl amine, rather than a LCB with attachment of a benzene ring [the ring and double bond equivalent (RDB) of a benzene ring is equal to 4].

**Fig. 4:**
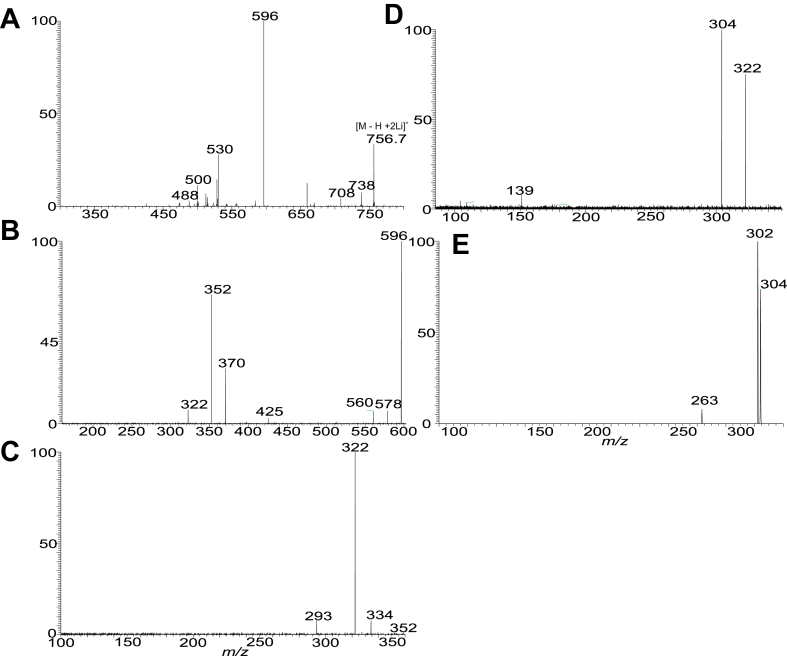
The MS^2^ spectrum of the [M – H + 2Li]^+^ ion of d20:4/βh17:0-glycerol phosphoryl ceramide at *m/z* 756 (corresponding to the [M – H]^-^ ion at *m/z* 742) (A), its MS^3^ spectrum of *m/z* 596 (756 → 596) (B), MS^4^ spectrum of *m/z* 352 (736 → 596 → 352) (C), MS^5^ spectrum of *m/z* 322 (736 → 596 → 352 → 322) (D), and MS^6^ spectrum of *m/z* 304 (736 → 596 → 352 → 322 → 304) (E). The above LIT MS^n^ spectra failed to provide sufficient structural information for location of the double bond position, due to lack of charge-remote fragmentation processes applicable for the assignments. LIT, linear ion trap.

**Scheme 5:**
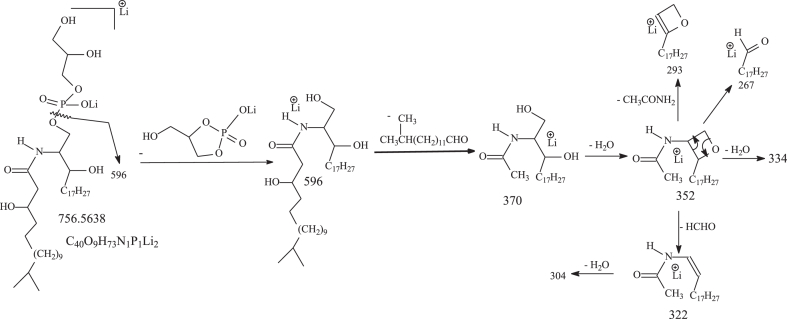
The fragmentation processes proposed for the [M – H + 2Li]^+^ ion of d20:4/βh17:0-Cer-PG at *m/z* 756.6. PG, phosphoglycerol.

To attempt to locate the position of the unsaturated bonds, we applied LIT MS^n^ on the corresponding lithiated adduct ions as previously described (33, 34). MS^2^ on the [M – H + 2Li]^+^ ion of d20:4/βh17:0-GPC at *m/z* 756 (corresponding to the [M – H]^-^ ion at *m/z* 742) (Fig. 4A), gave rise to a major ion at *m/z* 596, arising from loss of lithium glycerolphosphate (loss of [C_3_H_6_O_5_PO_3_HLi – H_2_O]; 160 Da) (Scheme 5). MS^3^ on the ion of *m/z* 596 (756 → 596; Fig. 4B) yielded major ion at *m/z* 370 arising from further loss of the βh17:0-FA substituent as an aldehyde (loss of C_14_H_29_CHO; 226 Da) and major ion at *m/z* 352 from further loss of H_2_O. The MS^4^ spectrum of *m/z* 352 (736 → 596 → 352; Fig. 4C) is dominated by the ion of *m/z* 322 arising from loss of HCHO (30 Da), along with ions at *m/z* 293 and 267 arising from cleavages of the N-acyl residues (Scheme 5), while the MS^5^ spectrum of *m/z* 322 (736 → 596 → 352 → 322; Fig. 4D) is dominated by *m/z* 304 (loss of H_2_O), which futher dissociates to *m/z* 302 by loss of H_2_ (Fig. 4E). The above results demonstrated that thermal degradations are the major fragmentation processes for the [M – H + 2Li]^+^ ion of d20:4/βh17:0-GPC upon being subjected to LIT MS^n^, resulting in insufficient structural information for assignment of the location of double bonds, which would otherwise require the charge-remote fragmentation processes for formation of informative ions applicable to the assignments (35).

### Characterization of the novel glycerol-bis-phosphoryldihydroceramide

**Fig. 5:**
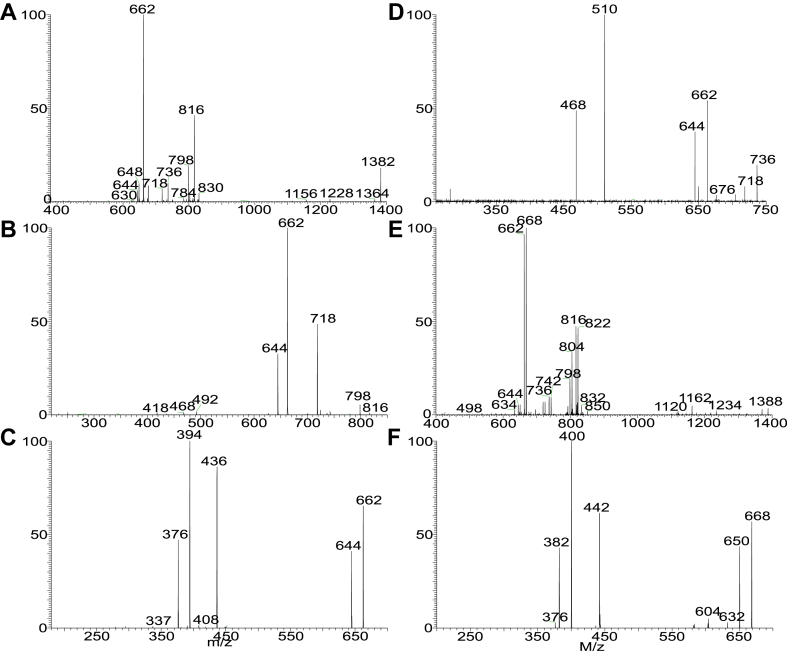
The MS^2^ spectrum of the [M – H]^-^ ion of new d19:0/βh17-DHC-P-G-P-d19:0/βh17-DHC lipid at *m/z* 1382 (A), its MS^3^ spectra of the ion of *m/z* 816 (1382 → 816) (B), of *m/z* 736 (1382 → 736) (D), MS^4^ spectra of *m/z* 662 (1382 →816 →662) (C). The MS^2^ spectrum of *m/z* 1388 (E), its MS^3^ spectra of *m/z* 662 (not shown; same as panel D), representing a d19:0/βh17:0-DHC-1-P anion, and of *m/z* 668 (F) equivalent to d20:4/βh17:0-Cer-1-P, indicating the presence of both a d19:0/βh17:0-DHC-1-P and a d20:4/βh17:0-Cer-1-P residues attached to the central glycerol (Scheme 6), leading to define a d19:0/βh17:0-DHC-PGP-d20:4/βh17:0-Cer structure in a new glycerol-bis-(phosphoryl)diceramide lipid with unique d20:4-LCB constituent. DHC, dihydroceramide.

**Scheme 6:**
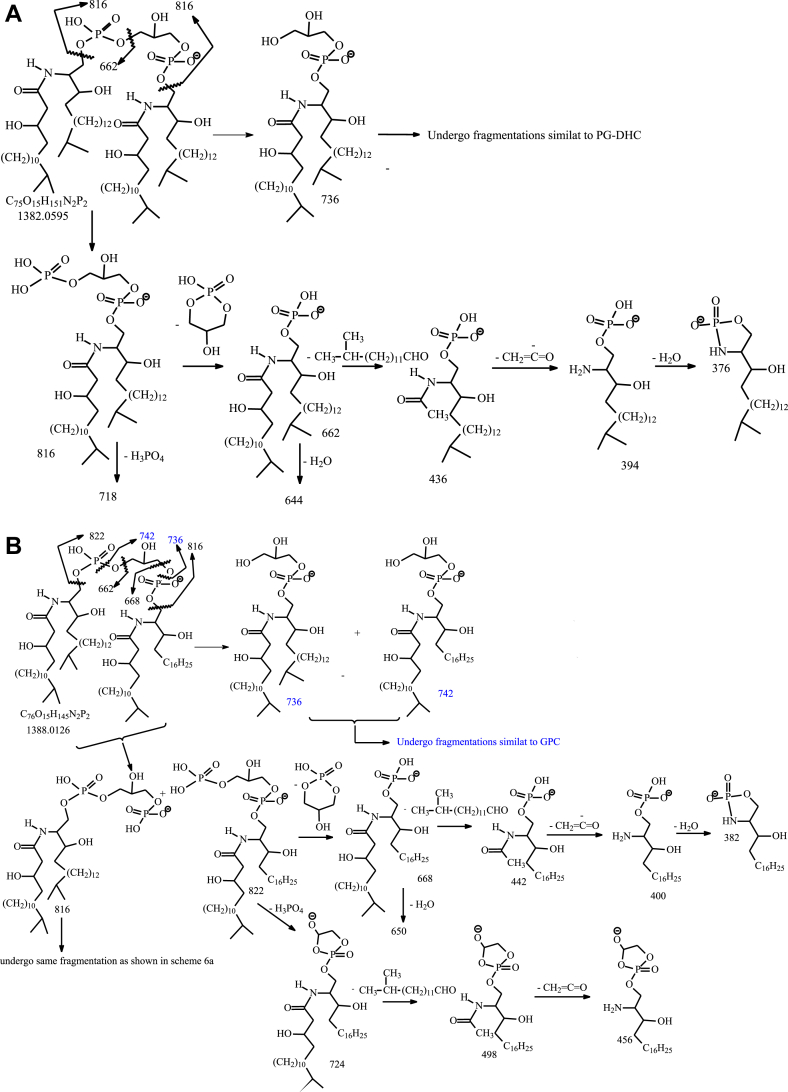
A: The fragmentation processes proposed for the [M – H ]^-^ ion of d19:0/βh17:0-Cer-PGP-d19:0/βh17:0-Cer at *m/z* 1382. B. The fragmentation processes proposed for the [M – H ]^-^ ion of d19:0/βh17:0-Cer-PGP-d20:4/βh17:0-Cer at *m/z* 1388.

A new family defined as glycerol-bis-(phosphoryldihydroceramide) (or bis-(dihydroceramide phosphoryl-glycerol) (DHC-PGP-DHC)) analogous to DHC-phosphorylinositol phosphate (PIP)-DHC recently reported for *B. vulgatus* was found. The lipid family exhibited a series of [M – H]^-^ ions ranging from *m/z* 1340.01287 to 1396.0758 with increment of 14.0156 Da (CH_2_) (Table 1) (supplemental Fig. S1E) and the corresponding [M – 2H]^-2^ ions ranging from *m/z* 669.5025 to 697.5339 with increment of 7.0078 Da (½ CH_2_) (See supplemental Table S1). A DHC-PGP-Cer subfamily in which the Cer residue consisting of polyunsaturated LCB and exhibiting the [M – H]^-^ ions at *m/z* 1359.9812.. and 1402.0279 with increment of a methylene (CH_2_) group, along with the corresponding [M – 2H]^-2^ ions (i.e., ions ranging from *m/z* 679.4867 to 700.5107) (supplemental Table S1) were also observed. The presence of DHC-PIP-Cer with polyunsaturated LCB is in accord with the observation of the polyunsaturated LCB–containing GPC subfamilies as seen earlier. Characterization of this novel DHC-PGP-DHC lipid family is exemplified by identification of the [M – H]^-^ ion at *m/z* 1382. The MS^2^ spectrum of *m/z* 1382 (Fig. 5A) contained prominent ions of *m/z* 816 arising from elimination of a DHC residue to form an DHC-PGP anion, together with ions of *m/z* 736/718 and 662/644 arising from further loss of phosphoric acid (HPO_3_H/H_3_PO_4_; 80/98 Da) and PG (HOP(O)O_2_C_3_H_5_OH/C_3_H_7_O_3_P(O)(OH)_2_; 154/172 Da), respectively (Scheme 6A). These fragmentation processes were supported by the MS^3^ spectrum of the ion of *m/z* 816 (1382 → 816; Fig. 5B), which are dominated by ions at *m/z* 718, 662, and 644. The MS^4^ spectrum of *m/z* 662 (1382 →816 →662; Fig. 5C) is identical to that of the [M – H]^-^ ion of d19:0/βh17:0-Cer-1-P; and the MS^3^ spectrum of the ion of *m/z* 736 (1382 →736; Fig. 5D) is similar to Fig. 2B arising from d18:0/βh17:0-GPC, thus, pointing to the presence of a d19:0/βh17:0-GPC substituent. The above results led to identification of a d19:0/βh17-DHC-P-G-P-d19:0/βh17-DHC structure (Scheme 6A).

In contrast, the MS^2^ spectrum of the ion at *m/z* 1388 (Fig. 5E) contained prominent ions at *m/z* 822 and 816, arising from losses of each of the two terminal ceramide residues, respectively; and consistent with the presence of abundant ions at *m/z* 668 and 662, respectively, arising from further loss of PG (Scheme 6B). The spectrum (Fig. 5E) also contained the *m/z* 736 and 742 ion pairs which are equivalent to d19:0/βh17:0-GPC and d20:4/βh17:0-GPC, respectively. The results demonstrated that the molecule contained both a d19:0/βh17:0-DHC and d20:4/βh17:0-Cer residues. This notion is further supported by MS^3^ on *m/z* 662 (not shown), which is identical to that shown in panel c, representing a d19:0/βh17:0-DHC-1-P anion, and on *m/z* 668 (Fig. 5F), which is equivalent to a d20:4/βh17:0-Cer-1-P. The above results define a d19:0/βh17:0-DHC-PGP-d20:4/βh17:0-Cer structure (Scheme 6B), a glycerol-bis-(phosphoryl)diceramide lipid with a DHC and a Cer with d20:4-LCB attached to the central glycerol.

**Fig. 6:**
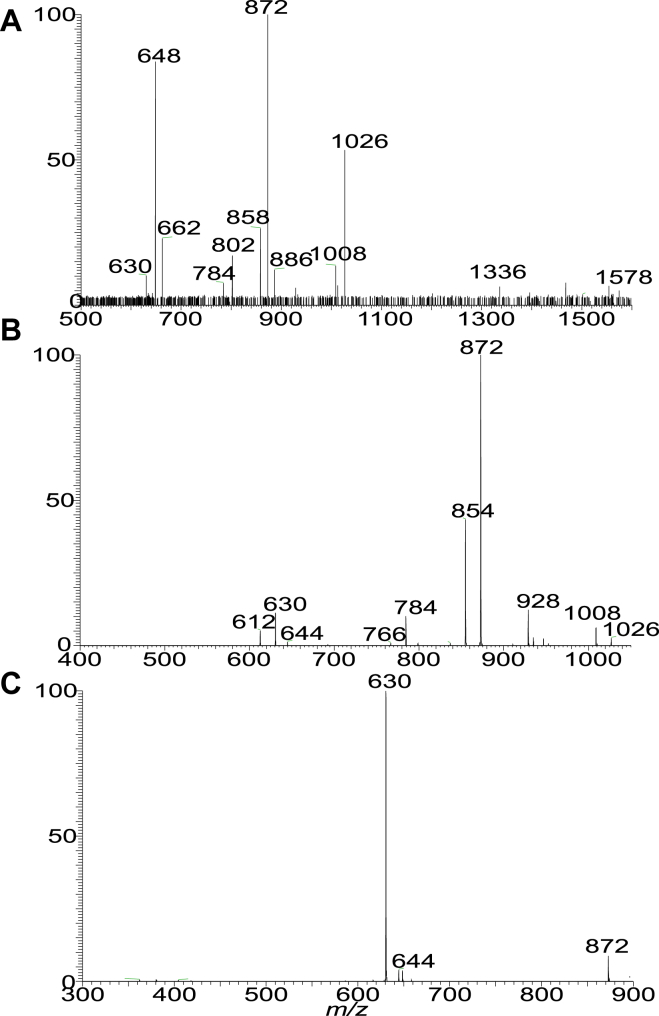
The MS^2^ spectrum of the [M – H]^-^ ion of d19:0/15:0-βh17-DHC-P-G-P-d19:0/βh17-DHC lipid at *m/z* 1578 (A), its MS^3^ spectrum of 1026 (1578 → 1026) (B) and MS^4^ spectrum of *m/z* 872 (1578 → 1026 → 872) (C). DHC, dihydroceramide.

**Scheme 7:**
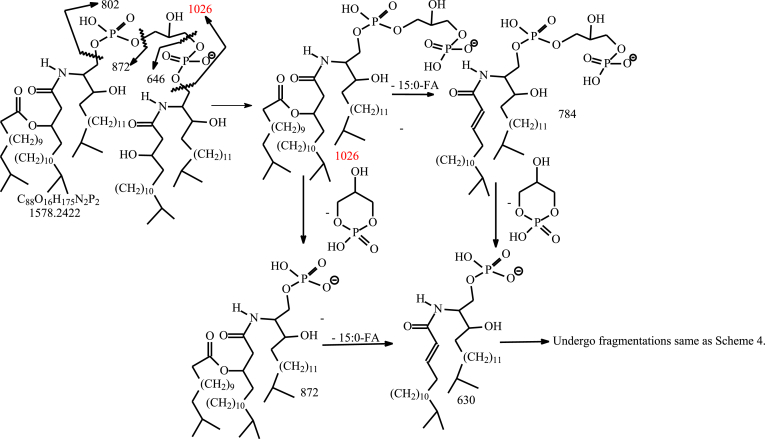
The fragmentation processes proposed for the [M – H]^-^ ion of d18:0/15:0-β17:0-Cer-PGP-d18:0/β17:0-Cer at *m/z* 1578.2 the branched LCB is according to the literature (ref 26). LCB, long-chain base.

HRMS also revealed a minor acylated DHC-PGP-DHC lipid subfamily (supplemental Fig. S1F), whose elemental compositions are C_15_H_30_O (224.2140 Da) heavier than the corresponding DHC-PGP-DHC lipids. For example, ion at *m/z* 1578.2422 is C_15_H_30_O (224.2140 Da) heavier than *m/z* 1354.0282, representing a d18:0/βh17:0-DHC-PGP-d18:0/βh17:0 lipid. As shown in Fig. 6A, the MS^2^ spectrum of the [M – H]^-^ ion at *m/z* 1578.24, contained the prominent ions at *m/z* 1026 and 802, arising from cleavage of ceramide residues (Scheme 7), and ions at *m/z* 872 and 648 representing a deprotonated d17:0/15:0-βh17:0-1-P and d17:0/βh17:0-1-P anions, respectively. The MS^3^ spectrum of the ion at *m/z* 1026 (Fig. 6B) is dominated by *m/z* 872 arising from loss of PG to form a deprotonated d17:0/15:0-βh17:0-1-P anion which further dissociated to *m/z* 630 by loss of the 15:0-FA side chain (the piggy back 15:0-FA chain). This latter fragmentation process is supported by MS^4^ spectrum of the ion at *m/z* 872 (Fig. 6C), which is dominated by *m/z* 630 (Fig. 6C). The results readily define a d17:0/15:0-βh17:0-DHC-PGP-d17:0/βh17:0-DHC structure.

### Characterization of the new 15:0/βhFA-GS-diacyl-PA lipid with unsaturated FA moieties

**Figure 7:**
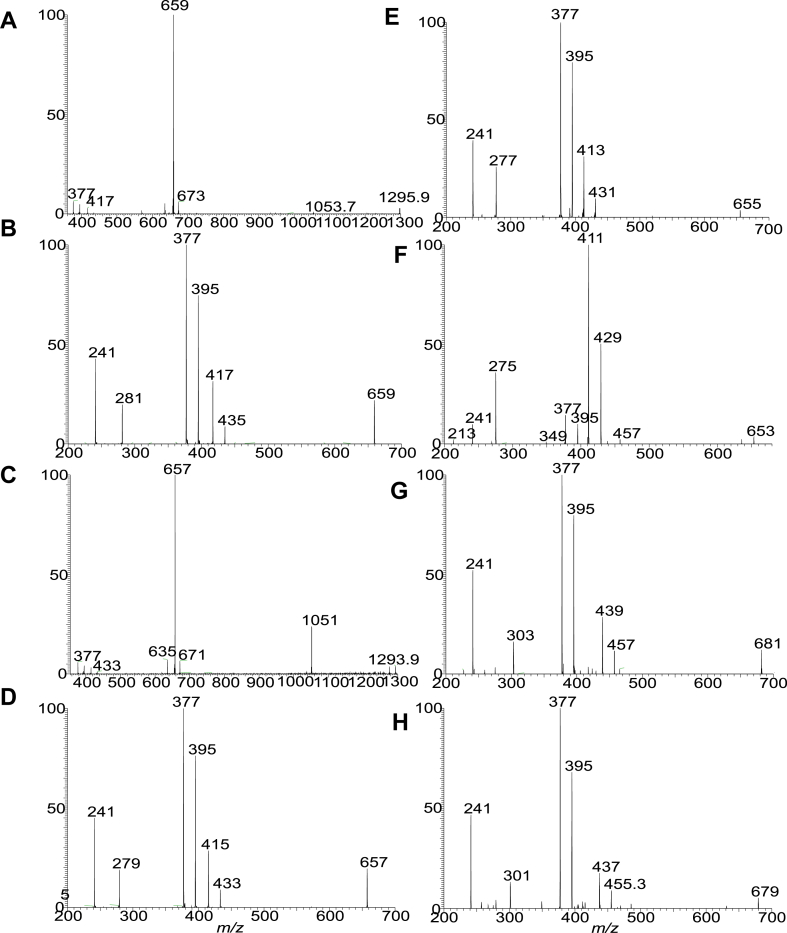
The LIT MS^n^ spectra that showed the GS-PA lipid species containing 1, (A and B), 2 (C and D), 3 (E), 4 (F and G), and 5 (H) unsaturated bond in sn-1 or sn-2 of the fatty acyl chains on PA. The MS^2^ spectra of the [M – H]^-^ ions at m/z 1295 (A) and 1293 (C) yielded a major ion equivalent to a deprotonated PA anion arising from loss of 15:0-βh17:0-GS residues. These [M – H]^-^ ions of diacyl-PA underwent the fragmentation processes identical to PA and gave rise to (1) ions from loss of sn-1 FA as acid and ketene, respectively; (2) ions from loss of sn-2 FA as acid and ketene, respectively; (3) the carboxylate anions (RCO_2_^-^) representing the sn-1 and sn-2 FA chains, respectively. The ions from losses of the FA chain at sn-2 are more abundant than the corresponding losses of the FA chain at sn-1, and the R_1_CO_2_^-^ is more abundant than the R_2_CO_2_^-^, leading to the assignment of the FA on the glycerol backbone. Therefore, MS^3^ spectrum of *m/z* 659 (1295.98 → 659) (B) defines the 15:0/18:1-PA moiety. MS^3^ spectrum of *m/z* 657 (1293.96 →657) (D) defines the 15:0/18:2-PA structure, MS^3^ spectrum of *m/z* 655 (1291.94 →655; panel E) defines 15:0/18:3-PA; MS^3^ spectrum of *m/z* 653 (1289.93 → 653; panel F) defines 18:4/15:0-PA; MS^3^ spectrum of *m/z* 681 (1317.96 → 681) (G) defines 15:0/20:4-PA, and MS^3^ spectrum of *m/z* 679 (1315.94 → 679) (H) defines 15:0/20:5-PA. Taken together, a 15:0-βh17:0-GS-15:0/18:1-PA (ion at 1295.98), 15:0-βh17:0-GS-15:0/18:2-PA (ion at 1293.96), 15:0-βh17:0-GS-15:0/18:3-PA (ion at 1291.94), 15:0-βh17:0-GS-18:4/15:0-PA (ion at 1289.93), 15:0-βh17:0-GS-15:0/20:4-PA (ion at 1317.96), and a 15:0-βh17:0-GS-15:0/20:5-PA (ion at 1315.94) structures can be assigned. GS, glycylserine; LIT, linear ion trap; PA, phosphatic acid.

The diacyl FA chains in the PA moieties of GS-PA lipids previously reported for *P. gingivalis* and *B. fragilis* are all saturated and branched (14, 27). Here, we report a new GS-PA subfamily in which the FA substituent at sn-1 or sn-2 is 18:1-, 18:2-, 18:3-. 18:4-, 20:4-, or 20:5-FA (supplemental Fig. S1G). The presence of the unsaturation bond in the FA chain is noticed by the findings that the elemental compositions (deduced from HRMS) of these GS-PA species possess 7.5, 8.5, 9.5, 10.5, and 11.5 RDB number, respectively, compared to the 6.5 RDB observed for the saturated GS-PA lipids. For example, the [M – H]^-^ ion at *m/z* 1295.9738 (calculated *m/z* for C_73_H_136_O_14_N_2_P: 1295.9736; RDB: 7.5) is 40.0316 Da (C_3_H_6_) heavier than ion at *m/z* 1255.9423 (C_70_H_130_O_14_N_2_P: 1255.9422; RDB: 6.5), a 15:0-βh17:0-GS-15:0/15:0-PA with branched 15:0-FA chain (Table 1). MS^2^ on the ion of *m/z* 1295.97 (Fig. 7A) gave rise to the major ion at *m/z* 659, arising from loss of 15:0-βh17:0-GS lipid residue. The MS^3^ spectrum of *m/z* 659 (Fig. 7B) is identical to that of 15:0/18:1-PA (36), leading to recognize a 15:0-βh17:0-GS-15:0/18:1-PA structure. Similarly, MS^2^ on the ion of *m/z* 1293.96 (Fig. 7C) gave rise to the major ion at *m/z* 657, arising from loss of 15:0-βh17:0-GS residue, MS^3^ on the ion of *m/z* 657 (Fig. 7D) yielded major ions at *m/z* 395 and 377, arising from loss of 18:2-FA substituent as ketene and acid respectively, and ions at *m/z* 415 and 433 arising from similar loss of 15:0-FA substituent. The results are consistent with the observation of the ions at *m/z* 241 (15:0-carboxylate) and *m/z* 279 (18:2-carboxylate anion). The ions from loss of 18:2-FA is more abundant than those from loss of 15:0-FA, indicating the presence of 15:0/18:2-PA (36), and thereby leading to define a 15:0-βh17:0-GS-15:0/18:2-PA structure. Similar results were seen for the MS^2^ spectrum of *m/z* 1291.9, which is dominated by *m/z* 655 (data not shown). The profile of the MS^3^ spectrum of *m/z* 655 (1291.9 → 655; Fig. 7E) is similar to panel d, pointing to a 15:0/18:3-PA substituent, and leading to define a 15:0-βh17:0-GS-15:0/18:3-PA structure. Interestingly, MS^2^ on the ion of *m/z* 1289.93 (calculated *m/z* for C_73_H_130_O_14_N_2_P: 1289.9265; RDB: 10.5) yielded a major ion at *m/z* 653, due to similar loss of 15:0-βh17:0-GS residue. However, the MS^3^ spectrum of *m/z* 653 (1289.93 →653; Fig. 7F) is dominated by ions at *m/z* 411 and 429 arising from loss of 15:0-FA as acid and ketene, respectively; while ions at *m/z* 439 and 457 arising from analogous losses of 18:4 FA substituent are less abundant. The results revealed an 18:4/15:0-PA structure (36). Therefore, a 15:0-βh17:0-GS-18:4/15:0-PA structure, in which the polyunsaturated FA chain is situated at sn-1, can be assigned. The MS^2^ spectrum of the [M – H]^-^ ion at *m/z* 1317.96 (calculated *m/z* for C_75_H_134_O_14_N_2_P: 1317.9578; RDB: 10.5) also undergoes similar loss of 15:0-βh17:0-GS to form major ion at *m/z* 681 (data not shown), which gave rise to major ions at *m/z* 395 and 377 by loss of 20:4 FA as ketene and acid respectively, along with an ion at *m/z* 303 representing a 20:4-carboxylate anion (1317.96 → 681; Fig. 7G). The spectrum also contained ions at *m/z* 467 and 439 arising from loss of the 15:0-FA as ketene and acid, respectively, and *m/z* 241, representing a 15:0-carboxylate anion. Again, the 377/395 ion pair is more abundant than the 437/455 pair, pointing to the presence of a 15:0/20:4-PA moiety (36), and leading to assignment of a 15:0-βh17:0-GS-15:0/20:4-PA structure.

The presence of GS-PA species with even more unsaturated bonds, for example, 11.5 RDB was seen by the [M – H]^-^ ion at *m/z* 1315.94, which is 2 H lighter than *m/z* 1317.96. The MS^2^ spectrum of *m/z* 1315.9, again is dominated by ion of *m/z* 679 (data not shown) arising from similar loss of 15:0-βh17:0-GS residue. The MS^3^ spectrum of *m/z* 679 (1315 → 679; Fig. 7H) is equivalent to that of 15:0/20:5-PA. Taken together, the results define a 15:0-βh17:0-GS-15:0/20:5-PA structure.

We applied HCD MS^n^ on the unsaturated FA-AMPP, which was formed by acid hydrolysis followed by derivatization with AMP+ reagent (See supplemental information 2). The HCD MS^2^ spectra clearly showed that the odd-chain saturated FA substituents including 15:0- and 17:0-FA are in both the iso and anteiso forms, while even-chain FAs such as 16:0-FA is a straight chain similar to those reported for *B fragilis* (27). For the unsaturated FA substituents, Δ^9^ 18:1, and Δ^9,12^ 18:2 structures were clearly identified, less clear were Δ^6,9,12^ 18:3, and Δ^5,8,11,14^ 20:4, and no assignment of the double position can be made for for 18:4 and 20:5-FA.

## Discussion

*B. fragilis* and *P. gingivalis* belong to the Cytophaga-Flavobacteria-Bacteroides phylum and share similar genomes (37). Using MS-based shotgun lipidomic approaches, we found that several lipid families including DHC-1-P and GS-PA are present in the membranes of both *B. fragilis* (27) and *P. gingivalis* (this study). Given their close relationship, it is not surprising that these two bacteria contain common lipids, yet key differences in the lipid structure and lipid families/subfamilies were found in their lipid repertoires. For example, the GS-PA lipid of *m/z* 1227.9109 ([M – H]^-^) observed for *B. fragilis* mainly represents a 15:0/βh16:0-GS-14:0/15:0-PA, while it represents a 15:0/βh17:0-GS-15:0/13:0-PA in *P. gingivalis*. The PA residues in GS-PA lipid in the *B. fragilis* group are all saturated and branched. On the other hand, *P. gingivalis* produces several previously unreported GS-PA lipid subfamilies whose PAs contain 1, 2, 3, 4, or 5 unsaturated bonds. In *B. fragilis*, both phosphatidylinositol (PI) and PI DHC (inositol phosphoryl ceramide (IPC)) lipids are the most prominent with GS-PA lipid family also being abundant, but PG DHC (GPC) and acylated GPC are absent. In contrast, *P. gingivalis* lacked PI and PI DHC (IPC) lipids, with PG DHC (GPC) and acylated GPC lipids being the most prominent. The observation of the new DHC-PGP-DHC lipid family and abundant GPC in *P. gingivalis* in the present study, and of the recent new DHC-PIP-DHC lipid family and the abundant IPC lipids exclusively found in *B. vulgatus* (27) is also interesting. Therefore, it may not be that far-fetched to speculate that G(PDHC)_2_ and I(PDHC)_2_ are formed by condensation of two molecules of GPC and GPI, respectively, similar to the pathway by which cardiolipins in bacteria are synthesized by condensation of two molecules of phosphatidylglycerols (38).

The observation of the new SL subfamily that consists of polyunsaturated LCB found in this study is also worth attention. Although further study to determine the unsaturation status, such as the location of double bonds is required, to our knowledge, ceramides with polyunsaturated bonds (i.e., ≥ 3) in LCB have not been reported.

Nichols and coworkers reported the involvement of purified DHC-PG and GS lipids in the proinflammatory secretory reactions in gingival fibroblasts (10, 11, 12). Whether the new lipid families/subfamilies found in this study are virulence determinants contributing to human diseases remains to be tested. Nevertheless, this study to envision the entire lipidome of *P. gingivalis* may provide a conceptual basis for further research to achieve a better understanding of the roles the new lipids in the oral pathogen may play in the development of periodontal and other human diseases.

## Data Availability

All data are contained within the manuscript.

## Supplemental data

This article contains supplemental data.

## Conflict of interest

The authors declare that they have no conflict of interests with the contents of this article.
