# Supplementary material for: Lipidomic analysis of Porphyromonas gingivalis reveals novel glycerol bisphosphoceramide, phosphatidyl-, and phosphoglycerol dipeptide lipid families
Source: J Lipid Res. 2023 Nov 2;64(12):100470. doi: 10.1016/j.jlr.2023.100470 (PMC10757044; doi:10.1016/j.jlr.2023.100470)
Supplement: Supplementary materials [file mmc1.docx]

**Supplemental information (1 and 2)**

Lipidomic analysis of *Porphyromonas gingivalis* reveals novel glycerol bisphosphoceramide, phosphatidyl- and phosphoglycerol dipeptide lipid families

Washington University School of Medicine

# Brian Kleiboeker^1^, Cheryl Frankfater^1^, Mary Ellen Davey^2^, and Fong-Fu Hsu, *^1^

^1^Mass Spectrometry Resource, Division of Endocrinology, Metabolism, and Lipid research,

Department of Medicine, Washington University School of Medicine, St. Louis, MO 63110. **^2^Department of Microbiology, The Forsyth Institute, Cambridge, MA 02142**

*To whom the correspondence should be addressed: Dr. Fong-Fu Hsu, Box 8127, Washington University School of Medicine, 660 S Euclid, St. Louis, MO 63110. Tel: 314-362-0056; Fax: 314-362-7641; e-mail: fhsu@wustl.edu.

**TABLE OF CONTENTS**

**Supplemental information 1 S3**

Figure S-1

*HR ESI mass spectra of each of the novel lipid families obtained from P. gingivalis. S3*

Table S1-1

*The HR ESI MS of the [M - 2H]^-2^ ions of Cer-PGP-Cer lipid family in P. gingivalis.….S4*

Table S1-2

*The HR ESI MS of the [M + Cl]^-^ ions of DHC lipid family in P. gingivalis…………...…S4*

**Supplemental information 2 s5**

**Characterization of the fatty acid substituents**

Table s2-1

HR *accurate mass measurements of the [M – H]^-^ ions of FA released from acid hydrolysis S7*

Table s2-2

HR *accurate mass measurements of the M^-+^ ions of the FA-AMPP derivatives S7*

Figure s2-1 a-c

*The ESI MS^2^ spectra of the M^-+^ ion of the branched FA-AMPP derivatives at m/z 409 (a),*

*437 (b) and at m/z 423 (c) for determination of the methyl branch,*

*S7*

Figure s2-2 a-d

*The ESI MS^2^ spectra of the M^-+^ ion of the polyunsaturated FA-AMPP derivatives at m/z*

*449 (a), 447 (b) 445 (c) and 471 for locating the unsaturated bonds, S7*

**Characterization of acylated epc**

Figure s3

*ESI MS^2^ spectrum of the [M – H]^-^ ion of d18:0/15:0-βh17:0-EPC at m/z 929 (a), its MS^3^ spectrum at m/z 687 (929 →687)(b), and MS^4^ spectrum at m/z 437 (c) (929 →687→ 437)(c). The proposed fragmentation pathways are shown in Panel d.. S8*

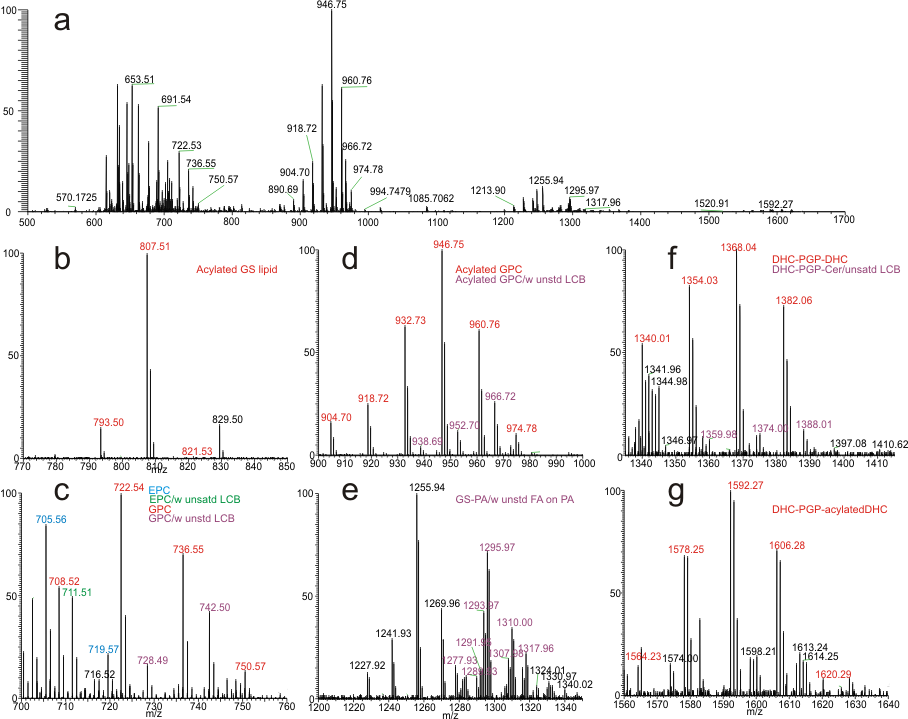


Fig S1 HR ESI mass spectra of the lipid families obtained from *P.* *gingivalis* outer membrane vesicle (OMV). (a) negative-ion full scan ESI-MS ranging from m/z 500 to 1700, which cover the entire lipidome including the novel lipid families that have not been reported previously. (b) the extended mass range from m/z 770 to 850 that showed the new acylated GS-lipids, (c) the mass range (700-760) shows EPC/EPC with unsaturated LCB and GPC/GPC with unsaturated LCB, (d) the mass range (900-1000) shows acylated GPC and novel acylated GPC with unsaturated LCB, (e) the mass range (1200-1350) shows GS PA and GS PA with unsaturated FA substituents on PA, (f) the mass range (1320-1420) shows the novel DHC-PGP-DHC and DHC-PGP-Cer/w unsaturated LCB, and (g) in the mass range (1560-1640) shows another novel DHC-PGP-acylated-DHC lipids. The detailed structures deduced by LIT MS^n^ are listed in Table 1.

| Table S1-1. The HR ESI MS of the [M - 2H]^-2^ of Cer-PGP-Cer lipid family in P. gingivalis | | | | | | |
| --- | --- | --- | --- | --- | --- | --- |
| m/z | Intensity | Relative | Theo. Mass | Deviation | RDB equiv. | Composition |
| [M - 2H]^-2^ |  | % | Da | mDa |  |  |
| 662.4946 | 7223.1 | 0.342 | 662.4948 | -0.23 | 3 | C71 H142 O15 N2 P2 |
| 662.9965 | 8897.5 | 0.423 | 662.9965 | -0.03 |  | C_70_ ^13^C_1_ H_142_ O_15_ N_2_ P_2_ |
| 669.5025 | 28080.3 | 1.332 | 669.5026 | -0.15 | 3 | C72 H144 O15 N2 P2 |
| 670.0040 | 24063.4 | 1.143 | 670.0043 | -0.25 |  | C_71_ ^13^C_1_ H_144_ O_15_ N_2_ P_2_ |
| 676.5104 | 77773 | 3.693 | 676.5105 | -0.07 | 3 | C73 H146 O15 N2 P2 |
| 677.0121 | 62288.9 | 2.958 | 677.0121 | 0.02 |  | C_72_^13^C_1_H_146_O_15_N_2_P_2_ |
| 683.5183 | 155642.5 | 7.389 | 683.5183 | 0.00 | 3 | C74 H148 O15 N2 P2 |
| 684.0200 | 119103.8 | 5.655 | 684.0200 | 0.00 |  | C_73_^13^C_1_H_148_O_15_N_2_P_2_ |
| 690.5261 | 151608.3 | 7.197 | 690.5261 | -0.03 | 3 | C75 H150 O15 N2 P2 |
| 691.0278 | 117138.6 | 5.562 | 691.0278 | -0.03 |  | C_74_^13^C_1_H_150_O_15_N_2_P_2_ |
| 697.5339 | 29600.8 | 1.404 | 697.5339 | -0.05 | 3 | C76 H152 O15 N2 P2 |
| 698.0356 | 20201.3 | 0.960 | 698.0356 | 0.05 |  | C_75_^13^C_1_H_152_O_15_N_2_P_2_ |
| 679.4867 | 6198.3 | 0.294 | 679.4870 | -0.29 | 7 | C74 H140 O15 N2 P2 |
| 679.9886 | 4918.3 | 0.234 | 679.9887 | -0.09 |  | C_73_^13^C_1_H_140_O_15_N_2_P_2_ |
| 686.4950 | 13038.8 | 0.618 | 686.4948 | 0.19 | 7 | C75 H142 O15 N2 P2 |
| 686.9963 | 13853.4 | 0.657 | 686.9965 | -0.21 |  | C_74_^13^C_1_H_142_O_15_N_2_P_2_ |
| 693.5029 | 21653.7 | 1.029 | 693.5026 | 0.26 | 7 | C76 H144 O15 N2 P2 |
| 694.0043 | 17168.6 | 0.816 | 694.0043 | -0.04 |  | C_75_^13^C_1_H_144_O_15_N_2_P_2_ |
| 700.5107 | 5369.1 | 0.255 | 700.5105 | 0.24 | 7 | C77 H146 O15 N2 P2 |
| 701.0120 | 2944.4 | 0.141 | 701.0121 | -0.06 |  | C_76_^13^C_1_H_146_O_15_N_2_P_2_ |

**Separation, chemical reaction, and mass spectrometric analysis toward structural definition of the fatty acid substituents in the lipid molecules**

**Experimental:**

*Acid hydrolysis and free fatty acid (FA) extraction.*

An aliquot of 100 uL of total extract (about 100 ug) in a vial, was dried under nitrogen and 1 mL CH_3_CN /37% HCl, 4/1 (v/v) was added, screw sealed with a Teflon liner cap, and vortexed at full speed for 10 seconds. The vial was heated at 90 °C for 2 hours. During heating, the vial was checked for seal every 30 min. After heating, the vial was cooled to room temperature, and 2 mL hexane were added, vortexed at full speed for 10 s, then centrifuged at 3000 x g for 3 min. The top layer containing fatty acids was transferred to another tube, dried under a stream of nitrogen, and FA-AMPP derivative was made as described below.

*Preparation of Fatty Acid-N-(4-aminomethylphenyl)pyridinium (FA-AMPP) derivatives*.

The acid hydrolysate was placed in a 20 mL glass tube, dried and FA-AMPP derivative was made with the AMP+ Mass Spectrometry Kit, according to the manufacturer’s instructions. Briefly, the dried sample was resuspended in 20 *μ*L of an ice-cold acetonitrile/DMF mixture [4:1 (v/v)], and 20 *μ*L of ice-cold 1 M 1-ethyl 3-[(dimethylamino)-propyl] carbodiimide hydrochloride (EDCI) in water was added. The vial was briefly mixed on a vortex mixer and placed on ice. To the vial, 10 *μ*L of 5 mM *N*-hydroxybenzotriazole (HOBt) solution and 30 *μ*L of 15 mM AMPP solution (in distilled acetonitrile) were added, vortexed for 30 seconds and heated at 65 °C for 30 min. After cooling to room temperature, 1 mL of water and 1 mL of *n*-butanol were added to the mixture. The final solution was vortexed for 1 min and centrifuged at 1200 x *g* for 3 min, and the top organic layer was transferred to another vial, and subjected to CID MS^2^ analysis.

*Tandem mass spectrometric analysis of FA as FA-AMPP derivative for identification of the fatty acid substituents in the molecules*

ESI HR mass measurement of the FAs from acid hydrolysis and the FA-AMPP derivatives, together with the higher energy collision induced dissociation (HCD) tandem mass spectra of the FA-AMPP were conducted on a Thermo LTQ Orbitrap velos at a resolution of 100,000 (at m/z 400 Da) as described in the main text.

**Results:**

HR ESI/MS analysis on the acid hydrolysate from lipid extract (Table S2-1) indicate the presence of the major fatty acids at m/z 241, 255, and 269, representing 15:0-, 16:0-, and 17:0-FA carboxylate anions, respectively, along with unsaturated fatty acid carboxylate anions at m/z 281 (18:1), 279 (18:2), 277 (18:3), 275 (18:4), 301 (20:5), and 303 (20:4). The results are consistent with the notion that FA substituents in the lipids consist of mainly the saturated/branch chains along with minor lipid families that contain polyunsaturated fatty acid chains such as the FA in GS-PA.

The presence of the various FA substituents is consistent with the observation of the corresponding M^+^ ions of FA-AMPP obtained by HR ESI/MS analysis on the acid hydrolysate-AMPP derivatives, which were seen, for examples, at m/z 409, 423, 445, and 447 (Table S2-2) corresponding to the carboxylate anions at m/z 241(15:0), 255 (16:0), 279 (18:2), 281 (18:1), respectively, in the acid hydrolysate (Table S2-1).

LIT MS^n^ analysis on the FA-AMPP derivatives indicated that the saturated fatty acyl chains such as 15:0-FA-AMPP (m/z 409; Figure s2a), 17:0-FA-AMPP (m/z 437; Fig s2b) are branched in the forms of ion- and anteiso with a ratio of about 1/1, while 16:0-FA-AMPP (m/z 423; Fig s2c), an even chain FA, is a straight chain. These results are similar to those previously reported for *B. fragilis* (1) and *P.gingivalis (2).*


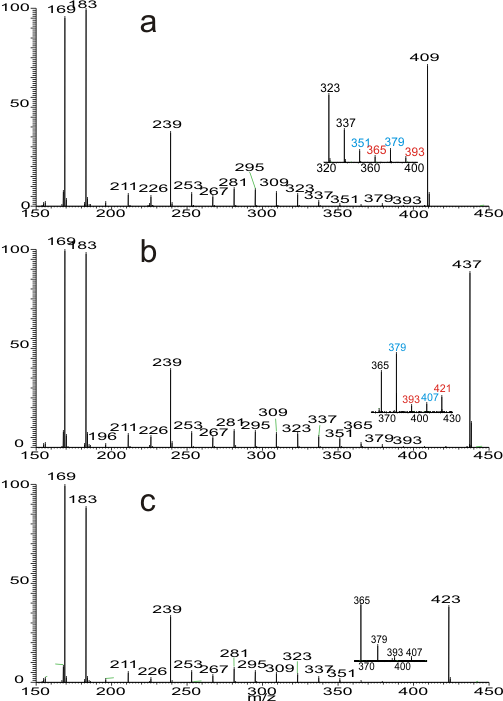


Fig S2-1 HCD MS2 spectra of the M+ ions of 15:0-FA-AMPP at m/z 409 (a), 17:0-AMPP at m/z 437 (b), and 16:0-AMPP at m/z 423 (c). In “insets” of Panels (a) and (b) showed ions from loss of CH_4_/[CH_4_+C_2_H_4_], and loss of C_2_H_6_/[C_2_H_6_+C_2_H_4_] pairs pointing to the iso- and anteiso-FA isomeric structures, respectively. In contrast, ions arising from cleavages of C-C bond were in descending order seen for m/z 423 (Panel c), indicating the presence of a straight chain 16:0-FA.

HCD MS^2^ analysis on the FA-AMPP derivatives was also used to locate the position of the double bonds of the fatty acid chain. The MS^2^ spectrum of the M^+^ ion of 18:1-AMPP at m/z 449 (Fig s3a) gave rise to ions at m/z 295 and 349 arising from allylic cleavage of the double bond at C-9, indicating the presence of oleic acid (Δ^9^ 18:1) (3). The HCD MS^2^ spectrum of the M^+^ ion of 18:2-AMPP at m/z 447 (Fig s3b) is nearly identical to that of linoleic acid (Δ^9,12^ 18:2) previously reported (4). However, HCD MS^2^ on the M^+^ ion of 18:3-AMPP at m/z 445 (Fig s3c) and of 20:4-FA APMM at m/z 471 (Fig s3d) did not yield quality spectra applicable for locating the double bonds mainly due to the fact that the molecular species are of low abundance (minor compounds) and the practical selected precursor ion window (1 Da) is surrounded many isobars that complicated the tandem mass spectra. As compared to the18:3- and 20:4-AMPP standard spectra previously, We speculate that they belong to γ-linolenic (Δ^6,9,12^ 18:3) and arachidonic acid (Δ^5,8,11, 14,^ 20:4), respectively (4). HCD MS^2^ spectra at m/z 469 from 20:5-AMPP and at m/z 443 from 18:4-AMPP (data not shown) gave rise to ions at m/z 169, 183, 226 and 239 that are signature ions for FA-AMPP, but were overwhelmed by many contaminated ions that prevent for recognizing the presence of double bonds.


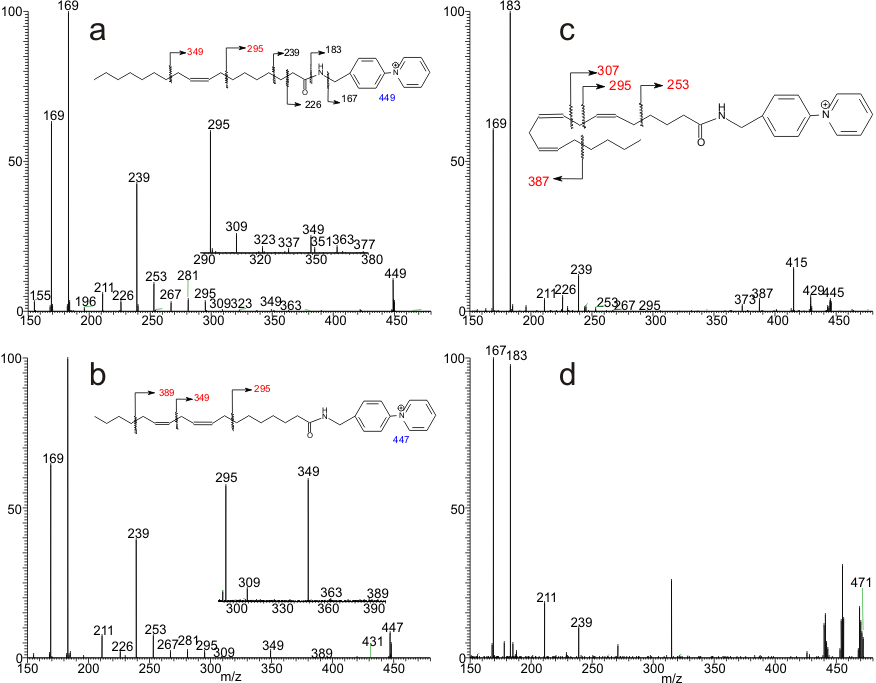


FigF FFig S2-2. The LIT HCD MS^2^ spectra of the M^+^ ions of 18:1-FA-AMPP at m/z 449, 18:2-FA-AMPP at m/z 447 (b), 18:3-FA-AMPP at m/z 445 (c), and 20:4-FA-AMPP at m/z 471 (d). Panel a (inset) showed the allylic cleavage of the C-C bond, leading to assignment of Δ^9^ 18:1-FA; Panel b (inset) illustrates the similar allylic cleavages around the homo-conjugated double bond at 9 and 12, leading to the Δ^9,12^ 18:2-FA structure. Panels (c) and (d) showed the similar fragmentation patterns as the Δ^6,9,12^ 18:3-FA and of Δ^5,8,11,14^ 20:4-FA standards, but lack the diagnostic ions applicable for locating the double bonds.

Fig s4-4. SIM shows slight differences in the abundance ratio of iso/anteiso isomers of 15:0- and 17:0-FA substituents among the four Bacteroides species in *B. fragilis* group


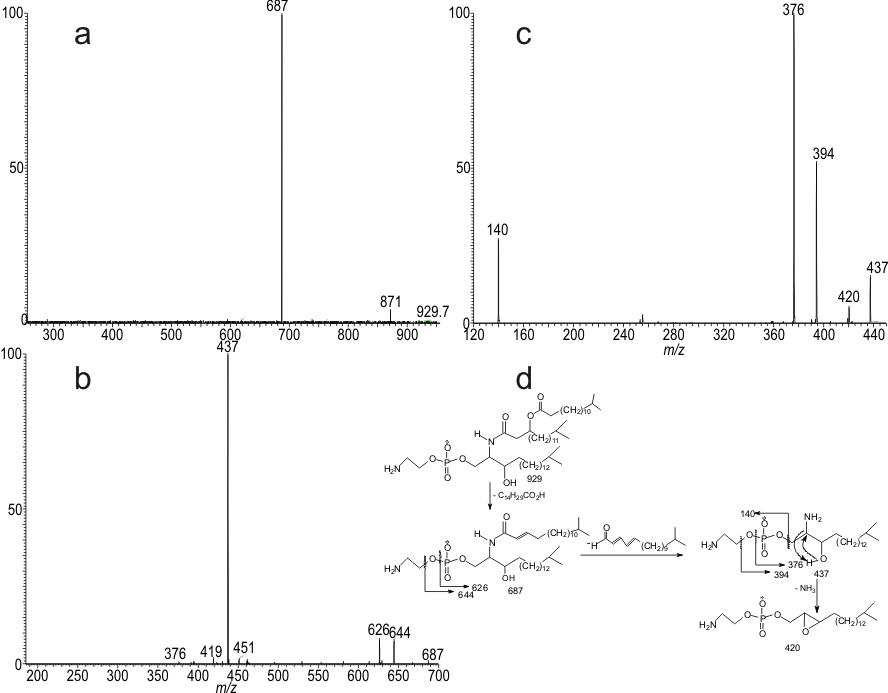


Fig S3. The ESI MS^2^ spectrum of the [M – H]^-^ ion of d18:0/15:0-βh17:0-EPC at m/z 929 (a), its MS^3^ spectrum at m/z 687 (929 →687) (b), and MS^4^ spectrum at m/z 437 (929 → 687→ 437) (c). The proposed fragmentation pathways leading to define the structure are shown in Panel d. The minor acylated EPC (DHC PE) lipid family was not reported in the earlier work by Nichols and coworkers [2].

References:

1. Frankfater, C. F., M. G. Sartorio, E. Valguarnera, M. F. Feldman, and F. F. Hsu. 2023. Lipidome of the Bacteroides Genus Containing New Peptidolipid and Sphingolipid Families Revealed by Multiple-Stage Mass Spectrometry. *Biochemistry* **62**: 1160-1180.

2. Nichols, F. C., R. B. Clark, M. W. Maciejewski, A. A. Provatas, J. L. Balsbaugh, F. E. Dewhirst, M. B. Smith, and A. Rahmlow. 2020. A novel phosphoglycerol serine-glycine lipodipeptide of Porphyromonas gingivalis is a TLR2 ligand. *J Lipid Res* **61**: 1645-1657.

3. Frankfater, C., X. Jiang, and F. F. Hsu. 2018. Characterization of Long-Chain Fatty Acid as N-(4-Aminomethylphenyl) Pyridinium Derivative by MALDI LIFT-TOF/TOF Mass Spectrometry. *J Am Soc Mass Spectrom* **29**: 1688-1699.

4. Yang, K., B. G. Dilthey, and R. W. Gross. 2013. Identification and Quantitation of Fatty Acid Double Bond Positional Isomers: A Shotgun Lipidomics Approach Using Charge-Switch Derivatization. *Anal. Chem.* **85**: 9742-9750.
